# BNC1 deficiency-triggered ferroptosis through the NF2-YAP pathway induces primary ovarian insufficiency

**DOI:** 10.1038/s41467-022-33323-8

**Published:** 2022-10-05

**Authors:** Feixia Wang, Yifeng Liu, Feida Ni, Jiani Jin, Yiqing Wu, Yun Huang, Xiaohang Ye, Xilin Shen, Yue Ying, Jianhua Chen, Ruixue Chen, Yanye Zhang, Xiao Sun, Siwen Wang, Xiao Xu, Chuan Chen, Jiansheng Guo, Dan Zhang

**Affiliations:** 1 Key Laboratory of Reproductive Genetics (Ministry of Education) and Department of Reproductive Endocrinology, Women’s Hospital, Zhejiang University School of Medicine, Zhejiang, 310006 China; 2 College of Computer Science and Technology, Zhejiang University, Zhejiang, 310027 PR China; 3 Department of Pathology, Women’s Hospital, Zhejiang University School of Medicine, Zhejiang, 310006 People’s Republic of China; 4 Department of Nutrition, Harvard T.H. Chan School of Public Health, Boston, MA 02215 USA; 5 Center of Cryo-Electron Microscopy, Zhejiang University, Hangzhou, Zhejiang China; 6 Clinical Research Center on Birth Defect Prevention and Intervention of Zhejiang Province, Hangzhou, 310006 China

**Keywords:** Genetics research, Infertility, Oogenesis, Apoptosis

## Abstract

Primary ovarian insufficiency (POI) is a clinical syndrome of ovarian dysfunction characterized by premature exhaustion of primordial follicles. POI causes infertility, severe daily life disturbances and long-term health risks. However, the underlying mechanism remains largely unknown. We previously identified a Basonuclin 1 (*BNC1*) mutation from a large Chinese POI pedigree and found that mice with targeted *Bnc1* mutation exhibit symptoms of POI. In this study, we found that BNC1 plays key roles in ovarian reserve and maintaining lipid metabolism and redox homeostasis in oocytes during follicle development. Deficiency of BNC1 results in premature follicular activation and excessive follicular atresia. Mechanistically, BNC1 deficiency triggers oocyte ferroptosis via the NF2-YAP pathway. We demonstrated that pharmacologic inhibition of YAP signaling or ferroptosis significantly rescues *Bnc1* mutation-induced POI. These findings uncover a pathologic mechanism of POI based on BNC1 deficiency and suggest YAP and ferroptosis inhibitors as potential therapeutic targets for POI.

## Abstract (web summary)

Primary ovarian insufficiency (POI) is a clinical syndrome of ovarian dysfunction that results in infertility. Here they show that BCN1 mutation results in premature ovarian follicle activation and atresia through dysregulation of ferroptosis.

## Introduction

Primary ovarian insufficiency (POI) is a clinical syndrome defined as premature exhaustion of the resting pool of primordial follicles before the age of 40 years and characterized by oligo-/amenorrhea for at least 4 months with elevated gonadotrophins (FSH level >25 IU/l on two occasions over 4 weeks apart). Approximately 1% of women under 40 years of age are affected^1,2^. POI causes severe daily life disturbances in these patients, including hot flashes, sweating, sleep disorders, vaginal dryness, depression, and compromised mental health^3^. Another major concern related to POI is the reproductive health impairment and even infertility among women of reproductive age. Potential etiologies for POI can be categorized as genetic, autoimmune, or iatrogenic. Although the immediate contributions of POI are unknown in most cases, this disorder generally arises from defects in primordial follicle pool formation, follicular recruitment/maturation, or follicular atresia^4–6^.

Past studies have focused on genes involved in POI pathogenesis. To date, genetic factors have been found to be associated with approximately 7% of POI cases^7^. Candidate gene screening has indicated that *BMP15, GDF9, NR5A1, FMR1, FIGLA*, and *PTEN* are closely related to POI. Defects in follicle recruitment and maturation have been studied in the context of POI. A lack of phosphatase and tensin homolog (PTEN) in oocytes can result in excessively activated primordial follicles and a subsequent decrease in the size of the primordial follicle pool^8,9^, while a lack of NOBOX accelerates postnatal oocyte loss and abolishes the transition from primordial to growing follicles^10^. However, the mechanism of follicular depletion is not well characterized. In our previous study, we found a large Chinese POI pedigree in which a heterozygous 5 bp deletion was identified in patients. The deletion led to frameshift mutation of *BNC1*, a premature TGA stop codon, loss of the nuclear localization signal (NLS) domain and three pairs of zinc finger (ZF) domains^11^. Basonuclin 1 (BNC1), a cell-type-specific transcription factor, is mainly expressed in the human basal keratinocytes of stratified epithelia, human testes, reproductive germ cells of mouse testes and ovaries and mouse one-cell embryos^12–14^. It has been found that knockdown of *BNC1* inhibits oocyte development. In addition, targeted *Bnc1* truncation mutation causes the phenotype of POI in mice^11^. We hypothesize that BNC1 deficiency may induce POI by perturbing oocyte development and thereby causing follicular atresia.

Oocyte death is closely connected with POI. Previous studies have revealed that apoptosis serves as a major type of cell death in oocyte loss. Primordial oocytes are most likely activated and then undergo apoptosis after cyclophosphamide injection, and their apoptosis can be inhibited by checkpoint 2 inhibitor treatment^15^. Severe DNA damage induces oocyte death upon TAp63α activation^16,17^. This evidence highlights the significance of oocyte death in the pathogenesis of POI.

Ferroptosis is a form of iron- and reactive oxygen species (ROS)-dependent regulated cell death. Lipid peroxidation products and lethal ROS have been found to accumulate during ferroptosis^18^. Recently, ferroptosis has become a popular research topic in the contexts of a variety of diseases, especially cancer^14,19^. Several recent findings reveal that cell density can affect the sensitivity of cells to ferroptosis via the Merlin (NF2)-YAP-Hippo pathway^20–22^. NF2 and/or LATS2 mutations lead to increased nuclear localization of YAP and promote proliferation and migration of cancer cells^23,24^. Some studies have reported that Hippo-YAP1 regulates primordial follicular activation^25,26^. However, activation of YAP can promote ferroptosis by upregulating several ferroptosis modulators, including ACSL4 and TFRC, which sensitize cancer cells to ferroptosis^20,27,28^. In patients with polycystic ovary syndrome (PCOS), iron-mediated mitophagy has been found to promote ferroptosis by activating TFRC/PINK1 signaling^29^. In addition, ovarian single-cell RNA sequencing has revealed an association between oocyte loss and ferroptosis around the perinatal period^30^. However, the role of ferroptosis in POI remains unknown.

In this work, we find that BNC1 is involved in lipid metabolism and maintaining redox homeostasis in oocytes. Dysfunction of BNC1 activates ferroptosis through the NF2-Hippo pathway in oocytes and ultimately leads to POI.

## Results

### *Bnc1* truncation mutation induces excessive activation and atresia of follicles

**Fig. 1:**
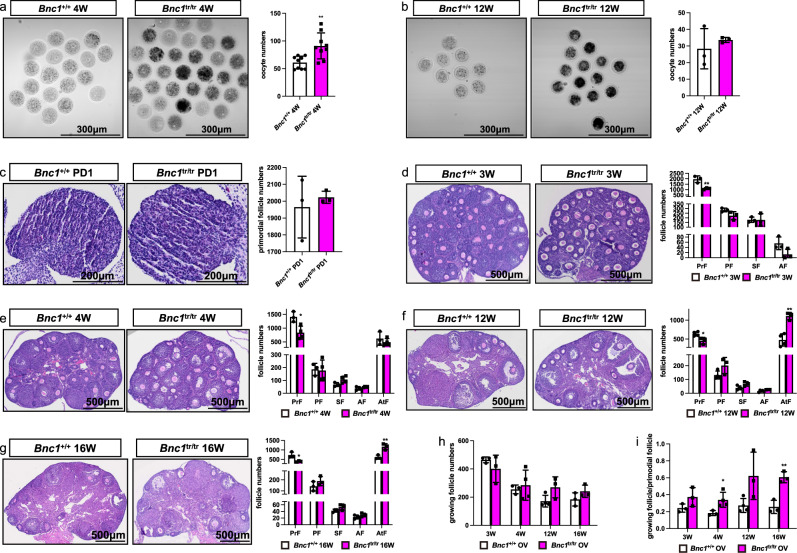
*Bnc1* mutation affects follicle development. **a** GV oocytes from *Bnc1*^+/+^ (*n* = 9) and *Bnc1*^tr/tr^ (*n* = 9) mice at 4 weeks old (*p* value = 0.0032). **b** GV oocytes from *Bnc1*^+/+^ (*n* = 3) and *Bnc1*^tr/tr^ (*n* = 3) mice at 12 weeks old (p value=0.4909). **c** Comparison of the numbers of primordial follicles (PrFs) of mice (*n* = 3) at PD1 (*p* value = 0.6174). **d** Comparison of the numbers of PrFs, primary follicles (PFs), secondary follicles (SFs) and antral follicles (AFs) of mice (n = 3) at 3 weeks old (*p* value = 0.0093 for PrFs, *p* value = 0.0903 for PFs, *p* value = 0.9641 for SFs, *p* value = 0.0804 for AFs). **e** Comparison of the follicle numbers of mice (*n* = 3) at 4 weeks old (*p* value = 0.0183 for PrFs, *p* value = 0.8694 for PFs, *p* value = 0.0769 for SFs, *p* value = 0.1996 for AFs, *p* value = 0.3926 for AtFs). **f** Comparison of the follicle numbers of mice (n = 4 for *Bnc1*^+/+^, n = 3 for *Bnc1*^tr/tr^) at 12 weeks old (*p* value = 0.0211 for PrFs, *p* value = 0.0910 for PFs, *p* value = 0.0560 for SFs, *p* value = 0.0571 for Afs, *p* value = 0.0021 for AtFs). **g** Comparison of the follicle numbers of mice (*n* = 3) at 16 weeks old (*p* value = 0.01810 for PrFs, *p* value = 0.2460 for PFs, *p* value = 0.1346 for SFs, *p* value = 0.2720 for AFs, *p* value = 0.0077 for AtFs). Scale bar = 500 μm. **h** Comparison of the growing follicle numbers of mice (*n* = 3) at 3, 4, 12, and 16 weeks old (*p* value = 0.3265 for 3 weeks old, *p* value = 0.6693 for 4 weeks old, *p* value = 0.0750 for 12 weeks old, *p* value = 0.1923 for 16 weeks old). **i** Comparison of the ratios of growing follicle numbers to PrFs of mice (*n* ≥ 3) at 3, 4, 12, and 16 weeks old (*p* value = 0.1352 for 3 weeks old, *p* value = 0.0341 for 4 weeks old, *p* value = 0.0592 for 12 weeks old, *p* value = 0.0040 for 16 weeks old). The error bars indicate the mean values ± SDs, unpaired *t* test, two-tailed, *p value < 0.05, **p value < 0.01 and ***p value < 0.001. Source data are provided as a Source Data file.

To determine the effect of *Bnc1* frameshift mutation on folliculogenesis, germinal vesicle (GV) oocytes were collected from 4- and 12-week-old mice superovulated with 10 IU of pregnant mare serum gonadotropin (PMSG). The dynamic changes in folliculogenesis in *Bnc1*^tr/tr^ mice (homozygous mutations) were analyzed and compared with those in wild-type (WT) *Bnc1*^+/+^ mice (Fig. 1a–b). We found an increase in oocyte retrieval in 4-week-old *Bnc1*^tr/tr^ mice compared to WT mice (Fig. 1a). However, the ovarian response of the *Bnc1*^tr/tr^ mice declined with increasing age (Fig. 1b). We hypothesized that the ovarian reserve of the *Bnc1*^tr/tr^ mice also declined with age. To test this hypothesis, we compared follicle numbers at postnatal day 1 (PD1) and weeks 3, 4, 12, and 16 in *Bnc1*^tr/tr^ and *Bnc1*^+/+^ mice. These data suggested that primordial follicle numbers in *Bnc1*^tr/tr^ mice were comparable to those in *Bnc1*^+/+^ mice at PD1 (Fig. 1c). At 3 weeks of age, *Bnc1*^tr/tr^ mice showed decreased numbers of primordial follicles and follicular overactivation, but apparent follicular atresia was not observed in *Bnc1*^tr/tr^ mice compared with *Bnc1*^+/+^ mice (Fig. 1d). At 4 weeks of age, compared with *Bnc1*^+/+^ mice, *Bnc1*^tr/tr^ mice also showed decreased numbers of primordial follicles and follicular overactivation (Fig. 1e). The phenotypes of 12-week-old and 16-week-old *Bnc1*^tr/tr^ mice were similar to those of 4-week-old mice. However, the numbers of atretic follicles increased significantly, while the primordial follicle depletion rate declined at 12 and 16 weeks of age (Fig. 1f, g). Overall, these data showed that follicles were overactivated (Fig. 1h–i) and that atretic follicle numbers increased with age in the mouse POI model with *Bnc1* truncation mutation (Fig. 1e–g).

### Conditional knockout of *Bnc1* in oocytes induces POI through excessive activation and atresia of follicles 

**Fig. 2:**
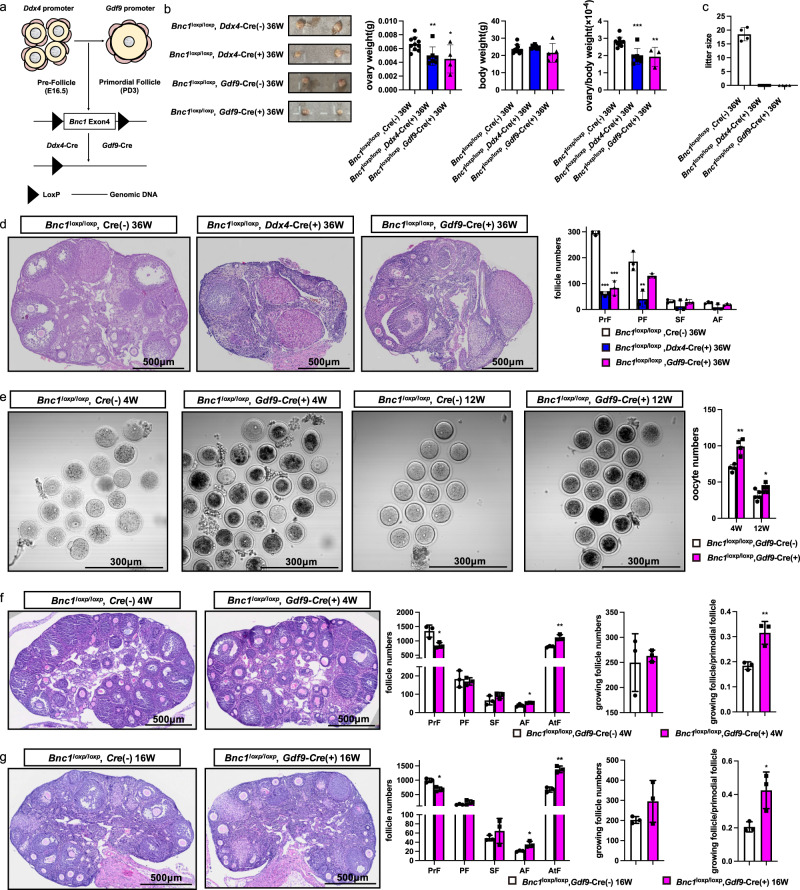
*Bnc1* affects follicle development by influencing oocytes. **a** Schematic representation of the deletion of *Bnc1* exon 4 in oocytes of pre-follicles, PrFs by using *Ddx4* and *Gdf9*-Cre transgenic. **b** Ovary weight, body weight and ovary weight/body weight ratios of *Bnc1*^loxP/loxP^, Cre (−) (*n* = 9), *Bnc1*^loxP/loxP^, *Ddx4*-Cre (+) (*n* = 7, *p* value = 0.0069 for ovary weight, *p* value = 0.2647 for body weight, *p* value = 0.0003 for ovary weight/body weight) and *Bnc1*^loxP/loxP^, *Gdf9*-Cre (+) (≥3, *p* value = 0.0179 for ovary weight, *p* value = 0.2835 for body weight, *p* value = 0.0029 for ovary weight/body weight) mice at 36 weeks. **c** Average litter sizes of *Bnc1*^loxP/loxP^, Cre (−) (*n* = 4), *Bnc1*^loxP/loxP^, *Ddx4*-Cre (+) (*n* = 5, *p* value < 0.0001) and *Bnc1*^loxP/loxP^, *Gdf9*-Cre (+) (*n* = 4, *p* value < 0.0001) female mice. **d** Comparison of follicle numbers of *Bnc1*^loxP/loxP^, Cre (−) (*n* = 3), *Bnc1*^loxP/loxP^, *Ddx4*-Cre (+) (*n* = 3, *p* value < 0.0001 for PrFs, *p* value = 0.0060 for PFs, *p* value = 0.2081 for SFs and *p* value = 0.0667 for AFs) and *Bnc1*^loxP/loxP^, *Gdf9*-Cre (+) (*n* = 3, *p* value = 0.0002 for PrFs, *p* value = 0.0620 for PFs, *p* value = 0.7357 for SFs and *p* value = 0.1135 for AFs) mice at 36 weeks. Scale bar = 500 μm. **e** GV oocytes obtained from *Bnc1*^loxP/loxP^, Cre (−) and *Bnc1*^loxP/loxP^, *Gdf9*-Cre (+) mice at 4 (*n* = 4) and 12 (*n* = 4) weeks old (*p* value = 0.0022 for 4 weeks old, *p* value = 0.0398 for 12 weeks old). **f** Comparison of follicle numbers of *Bnc1*^loxP/loxP^, Cre (−) and *Bnc1*^loxP/loxP^, *Gdf9*-Cre (+) mice at 4 weeks (*n* = 3, *p* value = 0.7145 for growing follicles, *p* value = 0.0091 for growing follicles/PrFs, *p* value = 0.0206 for PrFs, *p* value = 0.6640 for PFs, *p* value = 0.1959 for SFs, *p* value = 0.03219 for AFs and *p* value = 0.0065 for AtFs). **g** Comparison of follicle numbers of *Bnc1*^loxP/loxP^, Cre (−) and *Bnc1*^loxP/loxP^, *Gdf9*-Cre (+) mice at 16 weeks (*n* = 3, *p* value = 0.2046 for growing follicles, *p* value = 0.0287 for growing follicles/PrFs, *p* value = 0.0074 for PrFs, *p* value = 0.1718 for PFs, *p* value = 0.3858 for SFs, *p* value = 0.0219 for AFs and *p* value = 0.0011 for AtFs). Scale bar = 500 μm. The error bars indicate the mean values ± SDs, unpaired *t* test, two-tailed, *p value < 0.05, **p value < 0.01 and ***p value < 0.001. Source data are provided as a Source Data file.

To examine the role of *Bnc1* in oocytes, we deleted the *Bnc1* gene from mouse oocytes by crossing *Bnc1*^loxP/loxP^ mice with transgenic mice expressing growth differentiation factor 9 (*Gdf-9*) promoter-mediated Cre recombinase (referred to as *Gdf9*-Cre mice) and DEAD-box helicase 4 (*Ddx4*) promoter-mediated Cre recombinase (referred to as *Ddx4*-Cre mice) (Fig. 2a). According to the ovary weight/body weight ratio (i.e., the relative ovary weight) and ovary size, the ovaries of *Bnc1*^loxP/loxP^, *Ddx4*-Cre (+) and *Bnc1*^loxP/loxP^, *Gdf9*-Cre (+) mice were significantly smaller than those of WT mice at 36 weeks (Fig. 2b). *Bnc1*^loxP/loxP^, *Ddx4*-Cre (+) and *Bnc1*^loxP/loxP^, *Gdf9*-Cre (+) female mice were infertile (Fig. 2c). Histological and morphometric analyses revealed decreased follicle numbers in ovaries of *Bnc1*^loxP/loxP^, *Ddx4*-Cre (+) and *Bnc1*^loxP/loxP^, *Gdf9*-Cre (+) female mice compared with the ovaries of WT mice (Fig. 2d). This phenotype suggested that BNC1 played a role in the oocytes and functioned at the primordial follicle stage. Loss of BNC1 function could induce POI. GV oocytes were collected from 4- and 12-week-old mice superovulated with 10 IU of PMSG, which revealed dynamic changes in folliculogenesis in *Bnc1*^loxP/loxP^, *Gdf9*-Cre (+) mice compared with WT mice, and the ovarian response was consistent with that in *Bnc1*^tr/tr^ mice (Fig. 2e). We then counted follicles at 4 and 16 weeks in *Bnc1*^loxP/loxP^, *Gdf9*-Cre (−) and *Bnc1*^loxP/loxP^, *Gdf9*-Cre (+) mice. In comparison with *Bnc1*^loxP/loxP^, Cre (−) mice, *Bnc1*^loxP/loxP^, *Gdf9*-Cre (+) mice showed decreased numbers of primordial follicles, follicular overactivation and apparent follicular atresia at 4 and 16 weeks (Fig. 2f, g). Overall, these data showed that follicles were overactivated and that the numbers of atretic follicles were increased in oocyte-specific *Bnc1*-knockout mice.

### BNC1 deficiency in oocytes leads to follicular atresia through nonapoptotic cell death

**Fig. 3:**
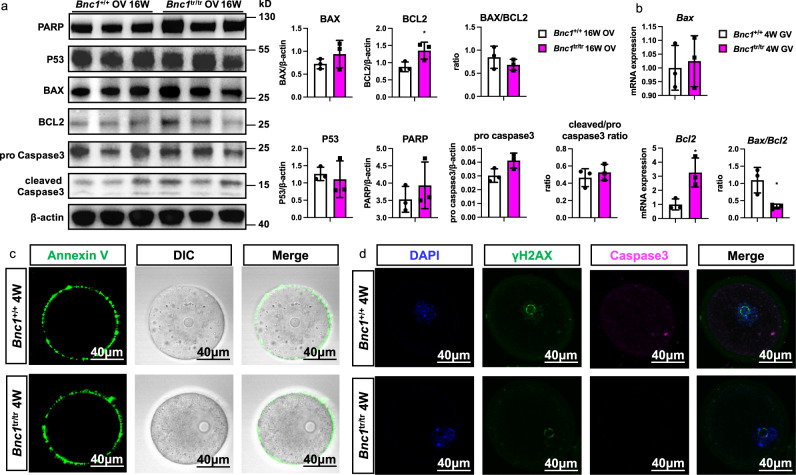
*Bnc1* mutation induces follicular atresia through nonapoptotic cell death. **a** Ovaries were obtained from *Bnc1*^+/+^ (*n* = 3) and *Bnc1*^tr/tr^ mice (*n* = 3) at 16 weeks old for western blotting (WB). The expression levels of P53, Bcl2, Bax, PARP, Caspase3 and cleaved-caspase3 are shown (*p* value = 0.6585 for P53, *p* value = 0.0457 for Bcl2, *p* value = 0.3090 for Bax, *p* value = 0.3467 for BAX/BCL2, *p* value = 0.0624 for Caspase3, *p* value = 0.4892 for cleaved-Caspase3/Caspase3 and *p* value = 0.4162 for PARP). **b** GV oocytes were obtained from *Bnc1*^+/+^ (*n* = 3) and *Bnc1*^tr/tr^ (*n* = 3) mice at 4 weeks old for real-time PCR. The mRNA expression of *Bax* and *Bcl2* is shown (*p* value = 0.7446 for *Bax*, *p* value = 0.0241 for *Bcl2* and *p* value = 0.0247 for *Bax*/*Bcl2*). **c** GV oocytes obtained from *Bnc1*^+/+^ and *Bnc1*^tr/tr^ mice at 4 weeks old were used for detection of early apoptosis (3 independent experiments with total oocyte numbers >30 oocytes). **d** GV oocytes obtained from *Bnc1*^+/+^ and *Bnc1*^tr/tr^ mice at 4 weeks old were used for detection of Caspase3 (CAS3) and the DNA damage marker γ-H2AX (3 independent experiments with total oocyte numbers >30 oocytes). The error bars indicate the mean values ± SDs, unpaired *t* test, two-tailed, *p value < 0.05, **p value < 0.01 and ***p value < 0.001. Source data are provided as a Source Data file.

Since apoptosis is involved in follicular atresia, we determined whether apoptosis contributed to ovary follicular atresia in the ovaries of *Bnc1*^+/+^ and *Bnc1*^tr/tr^ mice. However, the western blot analysis showed that the levels of apoptotic markers did not differ between *Bnc1*^+/+^ and *Bnc1*^tr/tr^ mouse ovaries (OV) at either 16 weeks (Fig. 3a) or 36 weeks (Supplementary Fig.1b). In addition, we assessed whether apoptosis occurred in oocytes. However, the results showed that *Bnc1* mutation and knockout in oocytes could induce antiapoptotic effects to some extent (Fig. 3b and Supplementary Fig. 1c). Annexin V staining also showed that there was no difference in early-stage apoptosis in *Bnc1*^tr/tr^ and *Bnc1*^loxP/loxP^, *Gdf9*-Cre (+) mouse oocytes compared with WT mouse oocytes (Fig. 3c and Supplementary Fig. 1d). Similarly, the DNA damage marker γH2AX and apoptosis marker caspase3 did not differ between the *Bnc1*^+/+^ and *Bnc1*^tr/tr^ mouse oocytes and the *Bnc1*^loxP/loxP^, *Gdf9*-Cre (−) and *Bnc1*^loxP/loxP^, *Gdf9*-Cre (+) mouse oocytes (Fig. 3d and Supplementary Fig. 1e). These results suggested that the process of follicular atresia in *Bnc1*^tr/tr^ and *Bnc1*^loxP/loxP^, *Gdf9*-Cre (+) mice was mediated not by apoptosis but rather by a nonapoptotic cell death pathway.

### Abnormal lipid metabolism and ferroptosis are involved in follicular atresia

**Fig. 4:**
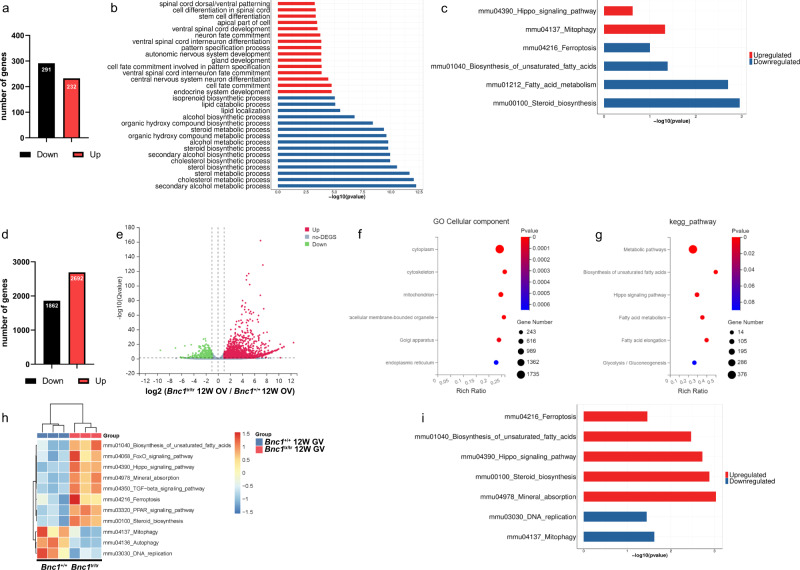
*Bnc1* mutation may induce follicular atresia through ferroptosis. **a** DEGs of *Bnc1*^+/+^ (*n* = 3) and *Bnc1*^tr/tr^ (*n* = 3) mouse ovaries. **b** KEGG analysis of *Bnc1*^+/+^ and *Bnc1*^tr/tr^ mouse ovary RNA-seq data (Top significantly (FDR  <  0.05, Benjamini–Hochberg p adjustment)). **c** GSVA of *Bn****c****1*^+/+^ and *Bnc1*^tr/tr^ mouse ovary RNA-seq data (Wilcox test p value). **d**, **e** DEGs of *Bnc1*^+/+^ (*n* = 3) and *Bnc1*^tr/tr^ (*n* = 3) mouse GV oocytes through Smart-seq2 (significantly (FDR < 0.05) up- and downregulated genes (|log2FC| > 0.5)). **f**, **g** GO and KEGG analyses of *Bnc1*^+/+^ and *Bnc1*^tr/tr^ mouse GV oocytes (Top significantly (*P* value < 0.05)). **h**, **i** GSVA of *Bnc1*^+/+^ and *Bnc1*^tr/tr^ mouse GV oocytes (Wilcox test *p* value). Three biological replicates per group were analyzed in duplicate.

To uncover the molecular mechanism by which *Bnc1* mutation led to follicular atresia, we conducted RNA sequencing (RNA-seq) of ovary tissues. There were 523 differentially expressed genes (DEGs) between the *Bnc1*^+/+^ and *Bnc1*^tr/tr^ mouse ovaries (Fig. 4a). Kyoto Encyclopedia of Genes and Genomes (KEGG) enrichment analysis and Gene set variation analysis (GSVA) showed disrupted lipid metabolism in the ovaries of *Bnc1*^tr/tr^ mice compared with those of WT mice (Fig. 4b, c). Moreover, the results of oocyte Smart-seq (with a switching mechanism at the 5ʹ end of the RNA transcript) were consistent with those of RNA-seq. There were 4554 DEGs between the *Bnc1*^+/+^ and *Bnc1*^tr/tr^ mouse oocytes (Fig. 4d, e). Gene Ontology (GO) analysis and KEGG enrichment analysis suggested the involvement of abnormal mitochondrial function and impaired lipid metabolism (Fig. 4f, g). Gene set variation analysis (GSVA) of the Smart-seq transcriptome data revealed several important pathways involved in follicular atresia in *Bnc1*^tr/tr^ mice, including the Hippo pathway and ferroptosis (Fig. 4h, i). Since mitochondria-mediated lipid peroxidation is crucial in ferroptosis^31,32^ and the Hippo pathway promotes ferroptosis^20,33^, these findings indicated that ferroptosis might be involved in the process of *Bnc1* mutation leading to POI.

### BNC1 deficiency-induced follicular atresia is modulated by ferroptosis

**Fig. 5:**
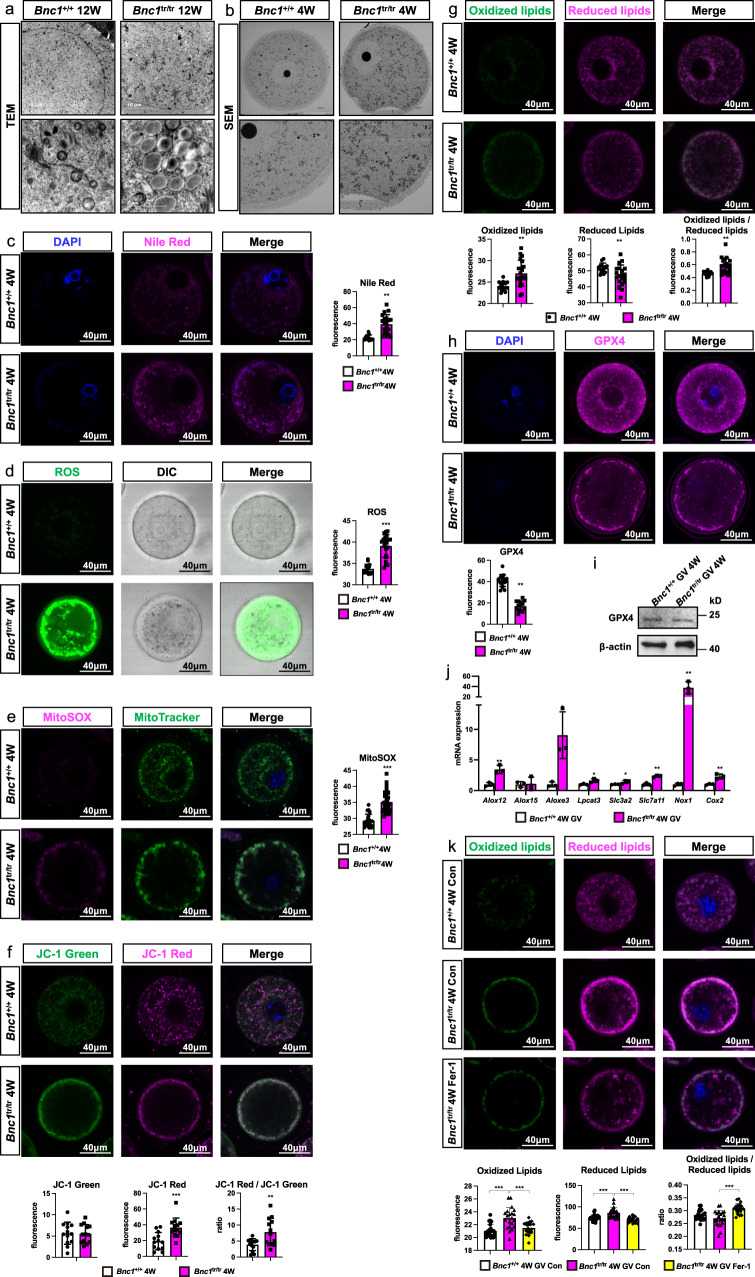
Oocytes affected by *Bnc1* mutation are more sensitive to ferroptosis. **a** TEM of mouse ovaries (*n* = 3). **b** SEM of GV oocyte (*n* = 1). **c** Nile red of GV oocytes (*p* value < 0.0001). **d** ROS in GV oocytes (*p* value < 0.0001). **e** MitoSOX and MitoTrack**e**r in oocytes (*p* value < 0.0001). **f** JC-1of GV oocytes (*p* value = 0.9010 for JC-1 green, *p* value = 0.0009 for JC-1 red and *p* value = 0.0055 for JC-1 red/green). **g** Lipid ROS in GV oocytes (*p* value = 0.0020 for oxidized lipids, *p* value = 0.0206 for reduced lipids and *p* value = 0.0013 for oxidized/reduced lipids). **h** GPX4 in GV oocytes (*p* value < 0.0001). **i** WB of GPX4 in GV oocytes (3 independent experiments). **j** RT–PCR of ferroptosis-associated markers in GV oocytes (*n* = 3) (*p* value = 0.0048 for *Alox12*, *p* value = 0.8962 for *Aloxe15*, *p* value = 0.0221 for *Aloxe3*, *p* value = 0.0307 for *Lpcat3*, *p* value = 0.0498 for *Slc3a2* and *p* value = 0.0010 for *Slc7a11*, *p* value = 0.0074 for *Cox2* and *p* value = 0.0055 for *Nox1*). **k** Lipid ROS in GV oocytes after Fer-1 treatment (for oxidized lipids *p* value *<* 0.0001 for *Bnc1*^tr/tr^ Con, *p* value = 0.0007, for *Bnc1*^tr/tr^ Fer-1, for reduced lipids *p* value = 0.0002 for *Bnc1*^tr/tr^ Con, *p* value < 0.0001, for *Bnc1*^tr/tr^ Fer-1, for oxidized to reduced lipids ratio *p* value = 0.0923 for *Bnc1*^tr/tr^ Con and *p* value < 0.0001 for *Bnc1*^tr/tr^ Fer-1). The error bars indicate the mean values  ± SDs, unpaired *t* test, two-tailed, 3 independent experiments with total oocyte numbers >30 oocytes, *p value < 0.05, **p value < 0.01 and ***p value < 0.001. Source data are provided as a Source Data file.

To address whether BNC1 deficiency triggers ferroptosis in oocytes, we assessed well-characterized morphological markers of ferroptosis in *Bnc1*-mutant mice. We performed transmission electron microscopy (TEM) of mouse ovaries and scanning electron microscopy (SEM) of mouse GV oocytes. We found that the *Bnc1* truncation mutation induced excessive lipid accumulation (Fig. 5a). In *Bnc1*^tr/tr^ mouse oocytes, abnormal mitochondrial distribution with abnormal aggregation was observed; an increased density of the mitochondrial membrane was also observed (Fig. 5b). Nile red staining of GV oocytes and mouse ovaries also confirmed that lipids accumulated in oocytes after *Bnc1* mutation and knockout (Fig. 5c, Supplementary Fig. 2a and Supplementary Fig. 3a, b). Furthermore, we examined mitochondrial function and the levels of oxidative stress markers, including ROS, MitoSOX, MitoTracker and JC-1. We found that ROS and MitoSOX levels were significantly increased in both *Bnc1*^tr/tr^ mouse oocytes and *Bnc1*^loxP/loxP^, *Gdf9*-Cre (+) mouse oocytes compared with WT mouse oocytes (Fig. 5d, e and Supplementary Fig. 3c, d). In addition, defects in mitochondrial distribution, with mitochondria localized beneath the cell membrane, were found in oocytes from *Bnc1*^tr/tr^ mice and *Bnc1*^loxP/loxP^, *Gdf9*-Cre (+) mice (Fig. 5e and Supplementary Fig. 3d). JC-1 staining showed that mitochondrial membrane potential was significantly increased in *Bnc1*^tr/tr^ and *Bnc1*^loxP/loxP^, *Gdf9*-Cre (+) mouse oocytes (Fig. 5f and Supplementary Fig. 3e), further suggesting that BNC1 deficiency was associated with ferroptosis^32^. Oocytes from *Bnc1*^tr/tr^ and *Bnc1*^loxP/loxP^, *Gdf9*-Cre (+) mice also exhibited significantly increased lipid ROS production (Fig. 5g and Supplementary Fig. 3f), a key driver in ferroptosis^2^. In addition, the decreased expression level of glutathione peroxidase 4 (GPX4) in *Bnc1*^tr/tr^ and *Bnc1*^loxP/loxP^, *Gdf9*-Cre (+) mouse oocytes further suggested that the *Bnc1* mutation and knockout sensitized oocytes to ferroptosis (Fig. 5h and Supplementary Fig. 3g). The level of GPX4 was also decreased in both the ovaries and oocytes from *Bnc1*^tr/tr^ mice (Fig. 5i, Supplementary Fig. 2b and Supplementary Fig. 3h). *Nox1* and *Cox2* were highly expressed (Fig. 5j and Supplementary Fig. 3i), which suggested the existence of dysregulated oxidative stress in *Bnc1*^tr/tr^ mouse ovaries and oocytes. The expression of ferroptosis marker genes, such as *Alox12*, *Aloxe3*, *Lpcat3*, *Slc3a2*, *Slc7a11*, was significantly abnormal in oocytes from *Bnc1*^tr/tr^ and *Bnc1*^loxP/loxP^, *Gdf9*-Cre (+) mice (Fig. 5j and Supplementary Fig. 3i). Furthermore, the ferroptosis agonist RLS3 significantly enhanced lipid peroxidation in *Bnc1*^tr/tr^ and *Bnc1*^loxP/loxP^, *Gdf9*-Cre (+) mouse oocytes (Supplementary Fig. 2c and Supplementary Fig. 3j). In addition, the ferroptosis antagonist ferrostatin-1 (Fer-1) partially reversed BNC1 deficiency-induced lipid peroxidation in *Bnc1*^tr/tr^ and *Bnc1*^loxP/loxP^, *Gdf9*-Cre (+) mouse oocytes (Fig. 5k and Supplementary Fig. 3k). Collectively, these results suggested that *Bnc1* mutation and knockout in oocytes induced ferroptosis.

### BNC1 deficiency triggers oocyte ferroptosis via the NF2-YAP pathway

**Fig. 6:**
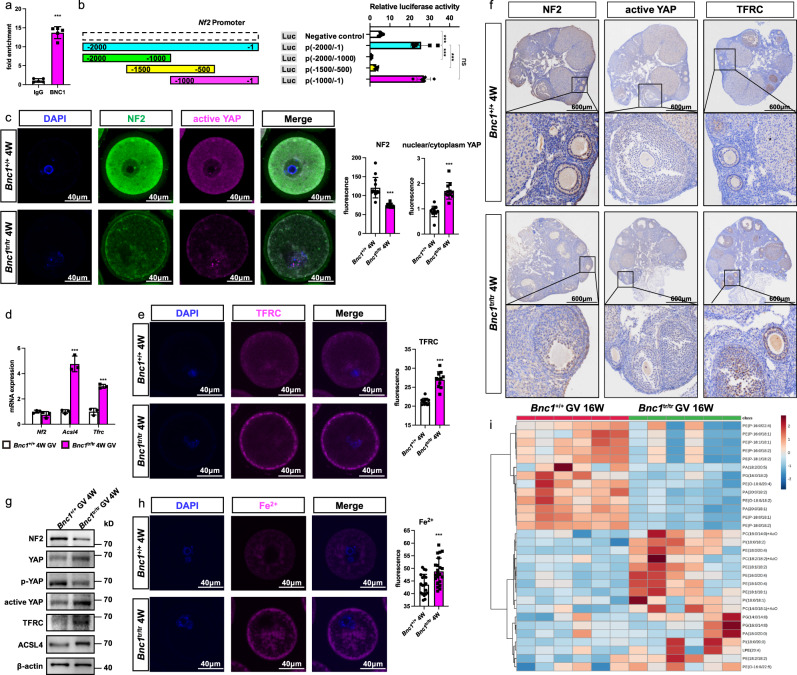
*Bnc1* truncation mutation sensitizes oocytes to ferroptosis by regulating NF2-YAP signaling. **a** ChIP–qPCR for BNC1 at *Nf2* promoter using mouse ovaries (*n* = 5, *p* value < 0.0001, 3 independent experiments). **b** Dual luciferase reporter assay of *Bnc1* at the *Nf2* promoter (*n* = 6, the error bars indicate the mean values ± SDs, unpaired *t* test, two-tailed, *p* value < 0.0001, 3 independent experiments). **c** NF2 and active YAP in GV oocytes at 4 weeks old (the error bars indicate the mean values ± SDs, unpaired *t* test, two-tailed, *p* value < 0.0001 for NF2, *p* value < 0.0001 for nuclear/cytoplasmic YAP, 3 independent experiments with total oocyte numbers >30 oocytes). **d** RT–PCR of *Nf2*, *Tfrc* and *Acsl4* in GV oocytes (*n* = 3) at 4 weeks old (*p* value = 0.3555 for *Nf2*, *p* value < 0.0001 for *Tfrc* and *p* value < 0.0001 for *Acsl4*). **e** TFRC of GV oocytes at 4 weeks old (the error bars indicate the mean values ± SDs, unpaired *t* test, two-tailed, *p* value < 0.0001, 3 independent experiments with total oocyte numbers >30 oocytes). **f** IHC of NF2, active YAP and TFRC in mouse ovaries at 8 weeks old (*n* = 3). **g** WB of NF2, p-YAP, active YAP, YAP, ACSL4 and TFRC in GV oocytes at 4 weeks old (3 independent experiments). **h** Fe^2+^ in GV oocytes at 4 weeks old (*p* value = 0.0010, the error bars indicate the mean values ± SDs, unpaired *t* test, two-tailed, *p* value < 0.0001, 3 independent experiments with total oocyte numbers >30 oocytes). **i** Heatmap of targeted lipidomics of *Bnc1*^+/+^ and *Bnc1*^tr/tr^ GV oocytes(*n* = 6). Source data are provided as a Source Data file.

To study the mechanism underlying the regulation of ferroptosis induced by BNC1 deficiency, we performed chromatin immunoprecipitation (ChIP) with sequencing (ChIP-seq). We found that NF2 might be a target gene of BNC1 (Supplementary Fig. 4). Knockdown of NF2 and activation of the Hippo pathway have been found to promote ferroptosis^20,22^. Thus, we determined whether BNC1 deficiency induced ferroptosis through the regulation of NF2 in oocytes. ChIP–qPCR (Fig. 6a) and luciferase activity (Fig. 6b) assays confirmed the binding of BNC1 to the promoter region of *Nf2*. Furthermore, a promoter deletion assay identified a fragment (bp −1 to −500) as the region required for BNC1 regulation of *Nf2* (Fig. 6b). Then, we examined the expression levels of NF2, active YAP, and transferrin receptor (TFRC) through immunofluorescence in oocytes and immunohistochemistry (IHC) in ovaries. We found that NF2 expression was significantly decreased in oocytes of *Bnc1*^tr/tr^ and *Bnc1*^loxP/loxP^, *Gdf9*-Cre (+) mice compared with those of WT mice (Fig. 6c, f and Supplementary Fig. 5a). Consistently, the nuclear localization of YAP and the expression of the downstream genes TFRC and ACSL4 were significantly increased in the oocytes of *Bnc1*^tr/tr^ and *Bnc1*^loxP/loxP^, *Gdf9*-Cre (+) mice (Fig. 6c, e–g and Supplementary Fig. 5a, b, d). The mRNA expression of *Nf2*, *Tfrc, Acsl4* and the protein expression of NF2, active YAP, p-YAP, YAP, ACSL4 and TFRC in oocytes from *Bnc1*^tr/tr^ and *Bnc1*^loxP/loxP^, *Gdf9*-Cre (+) mice showed the same trends as the above results (Fig. 6d, g and Supplementary Fig. 5c-d). TFRC has been identified as a key iron transporter on the plasma membrane^34^. Given the role of Fe^2+^ in lipid peroxidation and ferroptosis, we sought to determine the level of Fe^2+^ using the Fe^2+^-selective fluorescent probe FerroOrange. Confocal imaging revealed that FerroOrange intensity was significantly greater in oocytes of *Bnc1*^tr/tr^ mice than in oocytes of WT mice (Fig. 6h). Excessive iron-dependent peroxidation of polyunsaturated fatty acid (PUFA)-containing phospholipids (PUFA-PLs) induces ferroptosis^35–37^. ACSL4 is a key enzyme involved in PUFA-PL biosynthesis. Targeted lipidomics showed that phosphatidylethanolamine (PE) (18:1/18:2), PE (16:0/20:4), PE (18:2/18:2), PE (18:1/20:4), phosphatidylserine (PS) (14:0/20:4), and phosphatidylinositol (PI) (18:0/18:2) levels were higher in *Bnc1*^tr/tr^ mice than in WT mice (Fig. 6i and Supplementary Fig. 6a–f), suggesting that the levels of oocyte lipid substrates for peroxidation were increased after *Bnc1* mutation. In addition, increased lyso-phosphatidylethanolamine (LPE) (20:4) levels and decreased phosphatidic acid (PA) (20:0/18:1) levels were observed (Supplementary Fig. 6g, h); these molecules are related to ferroptosis^36,38^. These results suggested that BNC1 targeted NF2 to mediate the Hippo-YAP pathway, which was involved in the regulation of ferroptosis.

### Targeting NF2-Hippo/YAP signaling rescues *Bnc1* mutation-induced ferroptosis

**Fig. 7:**
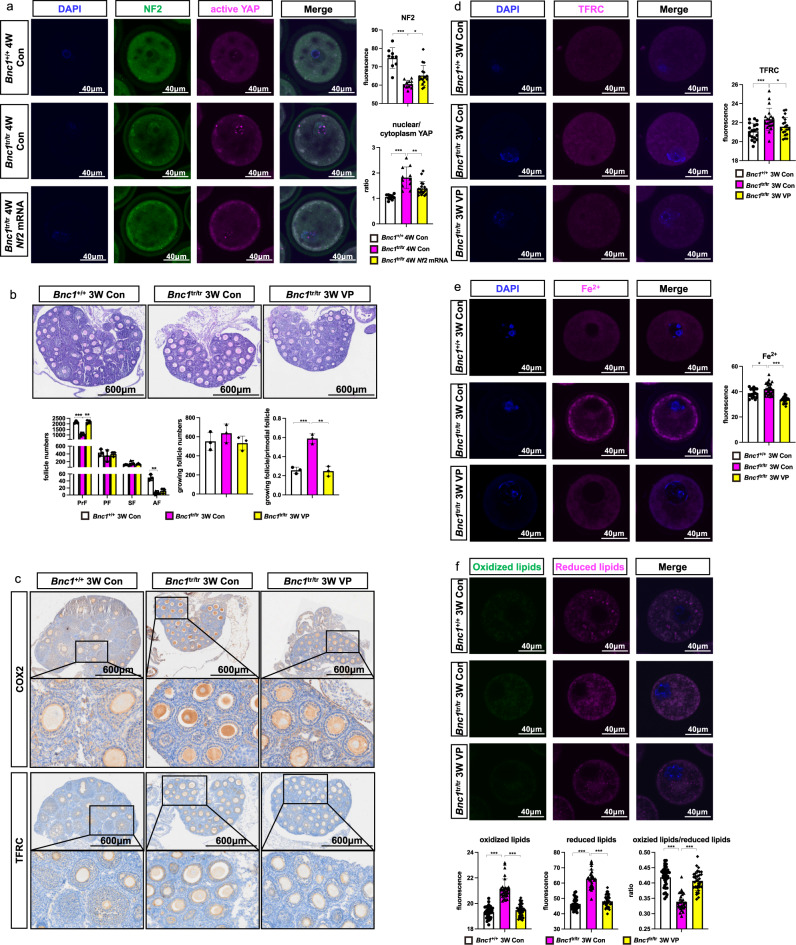
Targeting NF2-Hippo/YAP signaling pathway rescues *Bnc1* mutation induced ferroptosis. **a** Immunofluorescence of NF2 and active YAP in GV oocytes (*p* value < 0.0001 for NF2 of *Bnc1*^tr/tr^ Con, *p* value = 0.0120 for NF2 of *Bnc1*^tr/tr^ *Nf2* mRNA, *p* value < 0.0001 for nuclear/cytoplasmic YAP of *Bnc1*^tr/tr^ Con, *p* value = 0.0023 for nuclear/cytoplasmic YAP of *Bnc1*^tr/tr^ *Nf2* mRNA, 3 independent experiments with total oocyte numbers >30 oocytes). **b** HE staining and PrFs, PFs, SFs and AFs of *Bnc1*^+/+^ Con (*n* = 3), *Bnc1*^tr/tr^ Con (*n* = 3, *p* value = 0.0008 for PrFs, *p* value = 0.4975 for PFs, *p* value = 0.1942 for SFs, *p* value = 0.0019 for AFs, *p* value = 0.3379 for growing follicles and *p* value = 0.0007 for growing follicles/PrFs) and *Bnc1*^tr/tr^ VP (*n* = 3, *p* value = 0.0013 for PrFs, *p* value = 0.7104 for PFs, *p* value = 0.3955 for SFs, *p* value = 0.3796 for AFs, *p* value = 0.2134 for growing follicles and *p* value = 0.0012 for growing follicles/PrFs) mice at 3 weeks old. Scale bar = 600 μm. **c** IHC of COX2 and TFRC in *Bnc1*^+/+^ Con (*n* = 3), *Bnc1*^tr/tr^ Con (*n* = 3) and *Bnc1*^tr/tr^ VP (*n* = 3) mouse ovaries. **d** TFRC in mouse GV oocytes of *Bnc1*^+/+^ Con, *Bnc1*^tr/tr^ Con (*p* value = 0.0005) and *Bnc1*^tr/tr^ VP (*p* value = 0.0315) group at 3 weeks old. **e** Fe^2+^ concentration in *Bnc1*^+/+^ Con, *Bnc1*^tr/tr^ Con (p value=0.0115) and *Bnc1*^tr/tr^ VP (*p* value < 0.0001) mouse GV oocytes at 3 weeks old. **f** Lipid ROS in *Bnc1*^+/+^ Con, *Bnc1*^tr/tr^ Con (*p* value < 0.0001 for oxidized lipids, *p* value < 0.0001 for reduced lipids and *p* value < 0.0001 for oxidized/reduced lipids) and *Bnc1*^tr/tr^ VP (*p* value < 0.0001 for oxidized lipids, *p* value < 0.0001 for reduced lipids and *p* value < 0.0001 for oxidized/reduced lipids) mouse GV oocytes at 3 weeks old. The error bars indicate the mean values ± SDs, unpaired *t* test, two-tailed, 3 independent experiments with total oocyte numbers >30 oocytes. Source data are provided as a Source Data file.

Since *Bnc1* mutation induced ferroptosis through NF2/YAP signaling, we determined whether targeting NF2/YAP could reverse BNC1 deficiency induced ferroptosis. To further study the regulation of *Nf2* by BNC1, we microinjected *Nf2* mRNA into GV oocytes and cultured them for 24 h in vitro. Then, we examined the expression levels of NF2 and active-YAP through immunofluorescence in oocytes. We found that NF2 expression in oocytes of *Bnc1*^tr/tr^ mice was significantly increased after *Nf2* mRNA supplementation (Fig. 7a). Consistently, the level of YAP in nucleus was significantly decreased after *Nf2* mRNA injection (Fig. 7a). Next, we examined the effect of targeting YAP by its specific inhibitor verteporfin (VP). Mice were injected with either VP (100 mg/kg) or vehicle (corn oil) from the postnatal 7 days at 3-day intervals for 14 days. Then, we collected the ovaries at 3 weeks for follicle number counting. Compared with *Bnc1*^tr/tr^ mice, *Bnc1*^tr/tr^ mice injected with VP showed increased numbers of primordial follicles and decreased follicular overactivation, these results were comparable to those observed in *Bnc1*^+/+^ mice (Fig. 7b). The immunohistochemical assay showed that the expression of TFRC and COX2 was higher in *Bnc1*^tr/tr^ control mouse ovaries than in *Bnc1*^+/+^ control mouse ovaries (Fig. 7c). Intriguingly, VP treatment partially rescued TFRC and COX2 expression in *Bnc1*^tr/tr^ mouse ovaries (Fig. 7c). We subsequently detected TFRC expression and Fe^2+^ levels in GV oocytes, and found that TFRC expression was decreased in *Bnc1*^tr/tr^ mice treated with VP (Fig. 7d). Consistently, Fe^2+^ level was also decreased in VP-treated *Bnc1*^tr/tr^ mouse oocytes (Fig. 7e). Oocytes from *Bnc1*^tr/tr^ mice treated with VP exhibited significantly decreased lipid ROS level compared with those from *Bnc1*^tr/tr^ control mice (Fig. 7f). In vitro, knockdown of *BNC1* in ES-2 cells resulted in the down regulation of NF2, and phosphorylated YAP, and the upregulation of active-YAP, TFRC and ACSL4 (Supplementary Fig. 7a, b). These results further confirmed that BNC1 targeted the NF2 -Hippo-YAP-TFRC/ACSL4 pathway, deficiency of which induced ferroptosis.

### Inhibition of ferroptosis alleviates *Bnc1* mutation-induced POI in mice

**Fig. 8:**
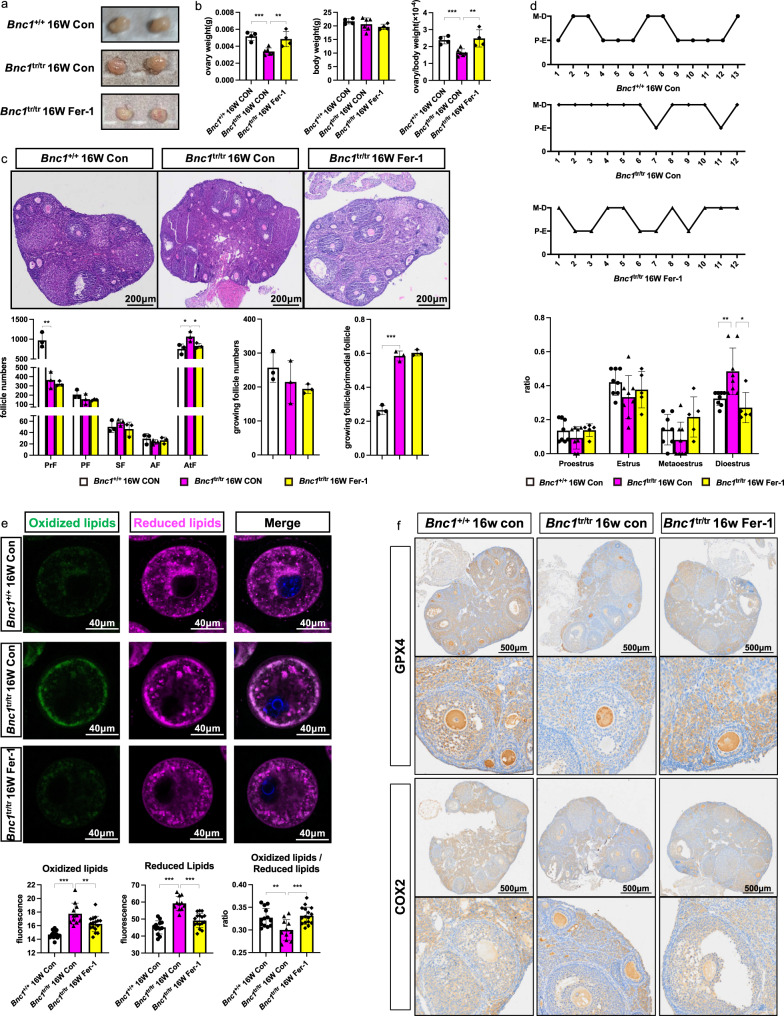
Fer-1 partially ameliorates the POI phenotype of the *Bnc1* truncation mutation. **a** Gross of *Bnc1*^+/+^ Con (*n* = 3), *Bnc1*^tr/tr^ Con (*n* = 3) and *Bnc1*^tr/tr^ Fer-1 (*n* = 3) mice at 16 weeks old. **b** The ovary weights, body weights and ovarian weight coefficients of *Bnc1*^+/+^ Con (*n* = 4), *Bnc1*^tr/tr^ Con (*n* = 6, *p* value = 0.0003 for ovary weight, *p* value = 0.4137 for body weight and *p* value = 0.0009 for ovary weight/body weight) and *Bnc1*^tr/tr^ Fer-1 (*n* = 4, *p* value = 0.0077 for ovary weight, *p* value = 0.4395 for body weight and *p* value = 0.0068 for ovary weight/body weight) mice at 16 weeks old. **c** HE staining of PrFs, PFs, SFs, AFs and AtFs of *Bnc1*^+/+^ Con (*n* = 3), *Bnc1*^tr/tr^ Con (*n* = 3, *p* value = 0.0073 for PrFs, *p* value = 0.3055 for PFs, *p* value = 0.3015 for SFs, *p* value = 0.3501 for AFs, *p* value = 0.0382 for AtFs, *p* value = 0.3957 for growing follicles and *p* value = 0.0002 for growing follicles/PrFs) and *Bnc1*^tr/tr^ Fer-1 (*n* = 3, *p* value = 0.4860 for PrFs, *p* value = 0.8158 for PFs, *p* value = 0.2028 for SFs, *p* value = 0.4977 for AFs, *p* value = 0.0471 for AtFs, *p* value = 0.6138 for growing follicles and *p* value = 0.4052 for growing follicles/PrFs) mice at 16 weeks old. Scale bar = 200 μm. **d** Estrous cycles of *Bnc1*^+/+^ Con (*n* = 8), *Bnc1*^tr/tr^ Con (*n* = 8, *p* value = 0.2186 for proestrus, *p* value = 0.1167 for estrus, *p* value = 0.2377 for metestrus and *p* value = 0.0073 for diestrus) and *Bnc1*^tr/tr^ Fer-1 (*n* = 5, *p* value = 0.1956 for proestrus, *p* value = 0.5312 for estrus, *p* value = 0.0536 for metestrus and *p* value = 0.0108 for diestrus) mice. **e** Lipid ROS in *Bnc1*^+/+^ Con, *Bnc1*^tr/tr^ Con (*p* value < 0.0001 for oxidized lipids, *p* value < 0.0001 for reduced lipids and *p* value = 0.0052 for oxidized/reduced lipids) and *Bnc1*^tr/tr^ Fer-1 (*p* value = 0.0083 for oxidized lipids, *p* value < 0.0001 for reduced lipids and *p* value = 0.0006 for oxidized/reduced lipids) mouse GV oocytes at 16 weeks old. 3 independent experiments with total oocyte numbers >30 oocytes). **f** IHC of GPX4 and COX2 in *Bnc1*^+/+^ Con (*n* = 3), *Bnc1*^tr/tr^ Con (*n* = 3) and *Bnc1*^tr/tr^ Fer-1 (*n* = 3) mouse ovaries. The error bars indicate the mean values ± SDs, unpaired *t* test, two-tailed, *p value < 0.05, **p value < 0.01 and ***p value < 0.001. Source data are provided as a Source Data file.

**Fig. 9:**
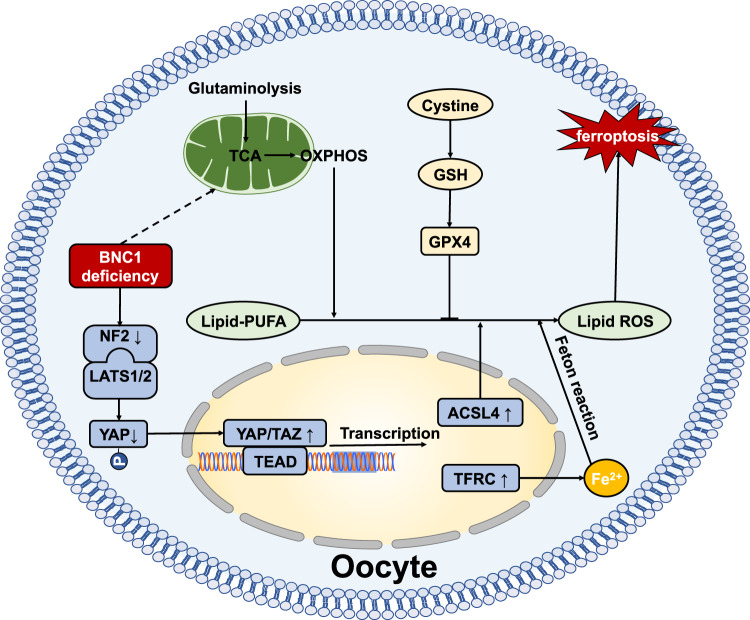
BNC1-Merlin-Hippo-YAP signaling -Ferroptosis axis. BNC1 directly regulates *Nf2* expression. BNC1 deficiency downregulates NF2 expression, which reduces YAP phosphorylation and promote YAP nuclear accumulation. YAP activation upregulates *Tfrc* and *Acsl4* expression, followed by increased iron uptake and enhanced lipid ROS production, which ultimately leads to oocyte ferroptosis.

Next, we explored whether ferroptosis in *Bnc1*^tr/tr^ mice directly induced POI and whether such impairment could be alleviated via the ferroptosis inhibitor Fer-1. We injected intraperitoneally (i.p.) Fer-1 into *Bnc1*^+/+^ mice and *Bnc1*^tr/tr^ mice every day and injected an equal dose of DMSO as a control. The ovary weight of *Bnc1*^tr/tr^ mice was lower (0.0034  ±  0.00042 g) than that of *Bnc1*^+/+^ mice (0.0052 ± 0.00051 g). Intriguingly, Fer-1 treatment significantly restored the ovary weight of *Bnc1*^tr/tr^ mice to normal levels (0.0049 ± 0.00090 g) (Fig. 8a, b). Moreover, the numbers of atretic follicles in *Bnc1*^tr/tr^ mice after Fer-1 treatment were comparable to those in the *Bnc1*^+/+^ control mice (Fig. 8c). In addition, on assessment of estrous cycle, *Bnc1*^tr/tr^ mice exhibited disordered estrous cycle with prolonged diestrus compared with *Bnc1*^+/+^ mice, but Fer-1 treatment partially improved this disorder and significantly shortened the duration of diestrus (Fig. 8d). Furthermore, the levels of oxidized lipids and reduced lipids were significantly lower in Fer-1-treated *Bnc1*^tr/tr^ mice than in *Bnc1*^tr/tr^ control mice, suggesting that lipid metabolism was improved with the inhibition of ferroptosis (Fig. 8e). The immunohistochemical assay showed that GPX4 expression was significantly lower and that COX2 expression was higher in *Bnc1*^tr/tr^ control mouse ovary follicles than in *Bnc1*^+/+^ mouse ovary follicles; however, Fer-1 treatment of *Bnc1*^tr/tr^ mice attenuated these differences in GPX4 and COX2 expression (Fig. 8f). Therefore, these results indicate an important role of ferroptosis in POI induced by BNC1 deficiency (Fig. 9) and suggest that block of ferroptosis might be a potential treatment for POI.

## Discussion

POI is caused by defective primordial follicle pool formation, follicular recruitment/maturation, and accelerated follicular atresia. It has long been considered that granulosa cell death pathways, including apoptosis, necrosis and autophagy, are involved in follicular atresia^39–44^. However, detailed information on oocyte death during POI remains limited. Ferroptosis, a recently recognized form of programmed cell death, has rarely been investigated in the context of the female reproductive system. BNC1, a transcription factor, is involved in oogenesis and folliculogenesis. We have found that the *Bnc1* truncation mutation leads to high FSH, reduced follicle numbers and fertility loss, which are typical phenotypes of POI^11^. In this study, we identified BNC1 as a factor that helps to maintain the ovarian reserve, deficiency of which results in POI by inducing oocyte ferroptosis. Regarding the mechanism, BNC1 deficiency in oocytes downregulates NF2, activates YAP, upregulates TFRC and ASCL4, and ultimately sensitizes oocytes to ferroptosis, which results in oocyte death and follicular atresia.

The expression of BNC1 in oocyte nuclei and the fact that POI results from BNC1 deficiency led us to investigate whether oocyte death contributes to follicular atresia^19^. We hypothesized that follicular atresia may be caused not by granulosa cell death but rather by oocyte death^45^. Oocyte-specific knockout of *Bnc1* induces the phenotype of POI, which suggests that *Bnc1* plays a crucial role in oocytes. *Bnc1* mutation leads to progressive fertility loss and testicular premature aging by inducing germ cell apoptosis in the testes^46,47^. However, *Bnc1* mutation results in POI not by mediating apoptosis. Intriguingly, ferroptosis induced by *Bnc1* mutation contributes to POI. These findings suggest that BNC1 functions in a tissue-specific manner.

During oogenesis, mitochondrial numbers increase rapidly, while the rate of oxygen consumption is maintained at a low level^48^. POI patients exhibit increases in both mitochondria mutations and ROS levels^49,50^. Many studies have shown that oxidative stress and mitochondrial dysfunction induce ovarian cell apoptosis to contribute to POI and ovarian aging^51,52^. Our findings are consistent with previous findings that some *Bnc1*-knockdown oocytes are morphologically and biochemically abnormal, evidenced by dark granules in the cytoplasm, and some oocytes degenerated whereas others could survive^53^. In this study, we found that the *Bnc1* mutation or oocyte-specific knockout model showed increased mitochondrial membrane potential and elevated ROS and MitoSOX levels in oocytes, which suggested the existence of an oxidative stress state in the oocytes. An increase in ROS level beyond a certain threshold may sensitize oocytes, particularly to lipid peroxidation induced ferroptosis, while low concentration of ROS may act as signal inducer. Intriguingly, the oocytes in *Bnc1*^tr/tr^ mice showed increased lipid peroxidation, which is a hallmark of ferroptosis. Our transcriptomic data also showed that ferroptosis is involved in *Bnc1*-induced POI. Several clinical lines of evidence support our findings. For example, women with POI exhibit abnormalities of lipid metabolism and metabolic profiles^54,55^. Dysregulation of follicle fatty acids is a potential driver of human POI, as free fatty acid treatment reduces the proliferation rate and induces obvious apoptosis signals in granulosa cells isolated from the ovaries^56^. Iron overload-induced ferroptosis may suppress the meiotic process during porcine oocyte maturation and lead to oxidative stress in each stage of the follicles, thus disturbing follicle development^57–59^. Here, we found that BNC1 is required for maintaining mitochondrial function and lipid metabolism in oocytes and deficiency of BNC1 triggers ferroptosis-dependent POI.

Targeting the Hippo pathway mediating ferroptosis has been shown to be a promising strategy in cancer therapy^20,21,33^. Inactivation of NF2 enables activation of the proto-oncogenic transcriptional coactivator YAP to promote ferroptosis via the upregulation of several ferroptosis modulators^20,22^. The Hippo-YAP/TAZ pathway can induce the expression of nicotinamide adenine dinucleotide phosphate (NADPH) oxidase 4 (NOX4), ALOXES, and SKP2, which contribute to YAP-promoted ferroptosis^21,33,60^. Given these findings, we focused on the NF2-YAP-Hippo pathway. We demonstrated that BNC1 directly targets NF2 expression, and that BNC1 deficiency leads to YAP activation and TFRC and ACSL4 upregulation. TFRC has been identified as a key iron transporter on the plasma membrane, and can lead to iron overload and lipid peroxidation. Upregulation of TFRC triggers ferroptosis^34^. ACSL4 is a key enzyme involved in PUFA-PL biosynthesis^35–37^. Our results show that increased ACSL4 promotes the accumulation of lipid peroxidation products. We also proved that in vitro, BNC1 regulated the NF2-YAP-TFRC/ACSL4 signaling axis in the ES-2 cell line. In breast cancer cell and renal cell carcinoma lines, loss of BNC1 expression increases cell motility, which is associated with a poorer prognosis^61,62^. In some studies, loss of function of NF2 has been reported to programmatically influences the redox imbalance that orchestrates malignant attributes of mammary/breast cancer^63,64^. These results support our finding that low NF2 expression in *Bnc1*-mutant mice results in a dysregulated cellular redox management system.

Here, we demonstrate that BNC1 is required for oocyte lipid metabolism and the redox system and affects the NF2-YAP signaling pathway. BNC1 deficiency results in ferroptosis-dependent follicular atresia. Our findings provide insights regarding the occurrence of ferroptotic oocyte death in POI and shed light on ferroptosis-targeted therapies for patients with POI.

## Methods

### Ethics approval for the research

The care and experimental procedures for the mice were in accordance with the Institutional Guidelines of the Animal Care and Use Committee (ACUC) and were approved by the ACUC of the Zhejiang University School of Medicine.

### Animal care

The mice were housed under controlled environmental conditions with free access to water and food. The mice were reared in standard conditions with controlled temperature (21–25 °C), humidity (40–70%) and the housing facility was illuminated between 7:00 am and 7:00 pm. All comparisons were made between littermates. All procedures were conducted according to the guidelines of the ACUC of the Zhejiang University School of Medicine Laboratory Animal Research Center. The experimental protocols were approved by the Regional Ethical Committee of Zhejiang University.

### Generation of *Bnc1*-targeted mutant mice and oocyte-conditional knockout mice

Mice with the targeted *Bnc1* mutation on a C57BL/6 J background were generated by the Nanjing Biomedical Research Institute of Nanjing University (Nanjing, China). The targeting vector was constructed with a 5 bp (CCGGG) deletion in exon 4 homologous to the identified human mutation, which leads to a frameshift downstream of the mutant site and a premature stop codon in the *Bnc1* transcript^11^. Male and female mice of the *Bnc1*^+/tr^ genotype were mated to produce *Bnc1*^+/+^, *Bnc1*^+/tr^ and *Bnc1*^tr/tr^ mice.

*Ddx4*-Cre and *Gdf9*-Cre knock-in mice were maintained on a C57BL/6 J background and obtained from Nanjing Biomedical Research Institute of Nanjing University (Nanjing, China). Dicer conditional null (flox) mice were maintained as a flox/flox stock on a C57BL/6 J background and generated by Innovative Cellular Therapeutics (Shanghai, China). *Ddx4*-Cre mice were genotyped by PCR using Cre primers (Cre forward: 5′ CACGTGCAGCCGTTTAAGCCGCGT 3′, Cre reverse: 5′ TTCCCATTCTAAACAACACCCTGAA 3′) that yielded a 240-bp DNA fragment. *Gdf9*-Cre mice were genotyped by PCR using Cre primers (Cre forward: 5′ TCTGATGAAGTCAGGAAGAACC 3′, Cre reverse: 5′ GAGATGTCCTTCACTCTGATTC 3′) that yielded a 500-bp DNA fragment. Dicer flox mice were genotyped with the PCR forward primer 5′ GACCTGGAAAGGCATGATGC 3′ and the PCR reverse primer 5′ GATCTCTCAGACTAGAAAGG 3′, yielding a 270-bp DNA fragment for the homozygous strain and a 177-bp DNA fragment for the WT strain. Initially, *Ddx4*-Cre and *Gdf9*-Cre heterozygotes were bred with *Bnc1*^flox/flox^ mice. The resulting male *Bnc1*^flox/+^, *Ddx4*-Cre and *Gdf9*-Cre mice were bred to female Cre (−), *Bnc1*^flox/flox^ mice to generate *Bnc1*^flox/flox^, *Ddx4*-Cre and *Gdf9*-Cre males and females. *Bnc1*^flox/flox^, *Ddx4*-Cre and *Gdf9*-Cre males were normal and fertile. Therefore, for the final cross, *Bnc1*^flox/flox^, *Ddx4*-Cre and *Gdf9*-Cre males were bred with, *Bnc1*^flox/flox^, Cre (−) females to generate *Bnc1*^flox/flox^, *Ddx4*-Cre and *Gdf9*-Cre females. *Bnc1*^flox/flox^, Cre (−) female littermates served as controls.

### Cell culture

The human ovarian cancer cell line ES-2 (CL-0079, Procell, China) was grown in McCoy’s 5A medium (C3020-0500, VivaCell) supplemented with 10% fetal bovine serum (FBS) (Serana, Germany), while the human embryonic kidney (HEK) 293 T cell line (ATCC, CRL-1573, USA) was cultured in high-glucose DMEM (Gibco, USA) supplemented with 10% FBS. All cells were cultured in a humidified atmosphere containing 5% CO_2_ at 37 °C, authenticated by Short tandem repeat (STR). The cells were plated into 6- or 24-well plates at a concentration of 100,000 cells per milliliter (20,000 cells/cm^2^). Cell growth was monitored, and treatment was initiated when cells reached 50% confluence.

### Gene silencing

Specific small-interfering RNAs (siRNAs) targeting human *BNC1* were generated by Gene Pharma (Shanghai, China). The sequences are listed in Supplementary Table 1. SiRNA was administered to cells with jetPRIME® Reagent (101000046, Polyplus transfection, France) according to the manufacturer’s instructions at 50 nM. The transfected cells were incubated at 37 °C and harvested at the indicated time points (24 h or 48 h) for the following assays.

### Histological analysis of ovarian tissues and counting of follicles

Ovarian tissues were collected at PD1 and at 3, 4, 12, and 16 weeks after the mice were born. The tissues were fixed in 4% paraformaldehyde, dehydrated in graded alcohol and xylene, and embedded in paraffin. The paraffin-embedded ovaries were serially sectioned at 5 μm thickness and stained with hematoxylin and eosin (HE) for morphological observation. As defined in a previous work^11^, the primordial, primary, secondary, and antral follicles were identified based on the well-accepted standards established by Pedersen and Peters were counted in sections at least 25 µm apart (each fifth section) spanning the entire ovary. The exact numbers of primordial follicles were determined by multiplying the raw numbers by a correction factor, which was 5 in this study.

### GV oocyte collection

To study ovarian responses to exogenous gonadotropins, female mice at 4 weeks and 12 weeks were injected i.p. with 10 IU of PMSG (Hong Kong Cen industrial, China). After 44–48 h, oocytes were collected from antral follicles by puncturing with a 26.5-gauge needle.

### mRNA synthesis and overexpression analysis

A T7 promoter vector encoding *Nf2* was generated by GuanNan Co., Ltd. (Hangzhou, China). For mRNA synthesis, the pcDNA3.1-*Nf2* (mouse) plasmids were linearized by BamHI-HF (R3136, NEB). Capped complementary RNAs (cRNAs) were made with an mMESSAGE mMACHINE T7 ULTRA Transcription Kit Invitrogen (AM1345, Thermo Fisher, USA) and purified with RNA extraction reagent (P1011, Solarbio, China). *Nf2* mRNA solution was injected into fully grown oocytes. To facilitate mRNA translation, oocytes were arrested in medium with 25 μmol/L 3-Isobutyl-1-methylxanthine (IBMX) for 24 h and then cultured in M16 medium for further experiments.

### Estrous cycle examination

A vaginal smear was taken every day with normal saline for 2–3 cycles of estrus. Approximately 0.1 ml of saline was drawn into the pipette, which was gently inserted into the vaginal canal. Then, the vaginal fluid was smeared on slides, stained with methylene blue after drying, and observed under a light microscope. The estrous cycle was classified into four stages, namely, the proestrus (P), estrus (E), metestrus (M), and diestrus (D) phases. The exact phase was determined by evaluating the major cell type^59^. Proestrus is characterized by low numbers of neutrophils, or large and nucleated epithelial cells, estrus is exhibited by predominantly anucleated keratinized epithelial cells, metestrus shows a combination of anucleated keratinized epithelial cells and neutrophils and diestrus is characterized by higher neutrophil numbers.

### Fertility test

Female mice were continuously mated with fertile male mice from the age of 8 weeks to 32 weeks (female:male = 1:1, *n* = 5 per genotype). The numbers of pups and litters were recorded.

### In vitro oocyte culture

GV oocytes from 4-week-old mice were treated with 5 µM RSL3 (T3646, Topscience, China) for 3 h in vitro. GV oocytes from 4-week-old mice were treated with 2 µM Fer-1 (S7243, Selleck Chemicals, USA) for 8 h in vitro.

### Immunofluorescence

Oocytes were fixed in 4% paraformaldehyde for 30 min at room temperature and washed 3 times with 1% bovine serum albumin (BSA) in phosphate-buffered saline (PBS). The oocytes were incubated in permeabilization buffer (1× PBS, 0.2% Triton-X 100 (Sigma–Aldrich, USA)) at 4 °C overnight and then blocked with 1% BSA in PBS for 1 h at room temperature. Then, the oocytes were incubated with primary antibodies at 4 °C overnight. After being washed three times with 1% BSA in PBS, the oocytes were incubated for 1 h at room temperature with secondary antibodies (Thermo Fisher) and DAPI (ab228549, Abcam, U.K.). Negative controls included parallel oocytes that were treated without primary antibodies. The oocytes were observed using a fluorescence microscope (Olympus IX81-FV1000).

The antibodies employed were as follows: anti-Caspase3 (1:50, #9664, Cell Signaling), anti-γH2AX (1:50, 05-636-I, Sigma–Aldrich), anti-NF2 (1:50, ab88957, Abcam), anti-active YAP (1:50, ab205270, Abcam), anti-TFRC (1:50, ab214039, Abcam), and anti-GPX4 (1:50, A13309, ABclonal, China).

### RNA isolation and sequencing

For preparation of ovary samples, total RNA was extracted with TRIzol reagent (Invitrogen, USA). The complementary DNA (cDNA) libraries for single-end sequencing were prepared using an Ion Total RNA-Seq Kit v2.0 (Life Technologies) according to the manufacturer’s instructions. The cDNA libraries were then processed for the Proton Sequencing process with an Ion PI Sequencing 200 Kit v2.0 (Life Technologies) by NovelBio Corp. Laboratory (Shanghai, China).

Single oocyte cDNAs were processed following a previously reported modified Smart-seq2 protocol^65,66^. Briefly, a single oocyte was transferred to 2 μl of cell lysis buffer with 1 μl of oligo-dT primer and 1 μl of dNTP mix (Fermentas). The tube was quickly vortexed to mix and incubated at 72 °C for 3 min. Then, the first-strand cDNA was reverse-synthesized using oligo(dT) and template-switching oligonucleotides (TSO). Reverse transcription was carried out by incubating the mixture at 42 °C for 90 min, 50 °C for 2 min, 42 °C for 2 min and 70 °C for 15 min. PCR preamplification was performed using the ISPCR primer. The reaction was incubated at 95 °C for 3 min; cycled 18 times at 98 °C for 20 s, 67 °C for 15 s, and 72 °C for 1 min; and then subjected to a final extension step at 72 °C for 5 min. Then, the cDNA was purified using VAHTS DNA Clean Beads N411. The primers were as follows: oligo(dT), 5′-AAGCAGTGGTATCAACGCAGAGTACT30VN-3′; TSO, 5′-AAGCAGTGGTATCAACGCAGAGTACATrGrG+G-3′; and ISPCR primer, 5′-AAGCAGTGGTATCAACGCAGAGT-3′. The cDNA library was constructed using a TruePrep DNA Library Prep Kit V2 for Illumina TD503 and a TruePrep Index Kit V2 for Illumina TD202. The Illumina® High-throughput sequencing platform (Novogene, China) was used.

### Mouse ovarian ChIP-seq, ChIP and qPCR

ChIP-Seq assays were performed as reported^47^. Each immunoprecipitation assay was performed using 25 mg of fresh ovary tissue with 2 µg anti-BNC1(ThermoFisher, PA5-85984) or anti-IgG control (2729, Cell Signaling, USA). The protocol of the SimpleChIP® Plus Enzymatic Chromatin IP Kit (Magnetic Beads) (9005, Cell Signaling) was followed. Briefly, 25 mg of fresh ovarian tissue was minced with a clean scalpel on ice. Then, the ovaries were fixed with 1.5% formaldehyde for in vivo crosslinking for 20 min at room temperature, which were terminated with glycine. Tissues were isolated into single cell suspensions using a Dounce homogenizer. Micrococcal Nuclease (10011, Cell Signaling) was added into each immunoprecipitation (IP) preparation, and the contents were mixed and incubated at 37 °C for 20 min with frequent agitation to digest the DNA to approximately 150-900 bp in length, followed by adding EDTA (7011, Cell Signaling) to stop digestion and then sonicated (Bioruptor Pico, Diagenode, Belgium) to disrupt the nuclear membrane. A total 10 µl of chromatin sample was removed as 2% input control before the antibodies were added. The IP samples were incubated at 4 °C with rotation overnight. Then, 30 μl of Protein G Magnetic Beads (9006, Cell Signaling) was added to each IP reaction, which was incubated at 4 °C for 2 h. After elution and reverse-crosslinking of chromatin from the antibodies/Protein G Magnetic Beads, the DNA was purified by spin columns. The primers used are listed in Supplementary Table 1.

### TEM and SEM

Ovaries collected from 12-week-old mice were fixed in 2.5% glutaraldehyde at room temperature for 2 h and then at 4 °C overnight. The ovaries were washed with PBS three times for 10 min. Then, the ovaries were fixed with 1% osmic acid for 1 h and washed with PBS three times for 10 min each. The ovaries were fixed with 2% uranyl acetate for 30 min; dehydrated with 50%, 70%, 90% and 100% ethanol for 10 min each; and washed with 100% acetone twice for 15 min each. The ovaries were embedded with the embedding agent acetone (1:1) for 2 h at room temperature, transferred to an embedding agent, embedded at 37 °C and polymerized. Photographs were taken with a Leica UC7 and cryogenic electron microscope (Tecnai G2 Spirit 120 kV).

Oocytes were fixed in 2.5% glutaraldehyde at room temperature for 2 h and then at 4 °C overnight. The oocytes were washed with PBS three times for 10 min each. The oocytes were then placed in an ice bath in the dark for 1.5 h after adding 2% osmic acid and 3% potassium ferric oxide (1:1). The oocytes were washed with ddH_2_O three times for 10 min each, transferred into 1% thiocarbohydrazide at 37 °C for 2 min and washed with ddH_2_O three times for 10 min each. The oocytes were then incubated away from light for 1 h after adding 2% osmic acid, washed with ddH_2_O three times for 10 min each, transferred into 1% uranyl acetate, and incubated at 4 °C overnight before being washed with ddH_2_O three times for 10 min each. Aluminum nitrate (0.66%) and aspartic acid were added, and the oocytes were incubated at 60 °C for 30 min. After the incubation, the oocytes were washed with ddH_2_O three times for 10 min each; dehydrated with 30%, 50%, 70%, 90% and 100% ethanol for 15 min each; transferred to acetone:ethanol (1:1) for 15 min; and then transferred to acetone for 20 min. The oocytes were transferred into 7:3 acetone:resin, 3:7 acetone:resin, and acetone overnight. Photographs were taken via SEM (Teneo VS, Thermo Fisher).

### Confocal microscopy of mouse oocytes

Oocytes were incubated with fluorescent dyes diluted with M16 medium (M7292, Sigma–Aldrich), including Reactive Oxygen Species Assay Kit (S0033S, Beyotime, China), MitoTracker® Green FM (40742ES50, Yeasen), MitoSOX Red Mitochondrial Superoxide Indicator (40778ES50, Yeasen), JC-1 (40705ES03, Yeasen), BODIPY™ 581/591 C11 (D3861, Thermo Fisher), FerroOrange (F374, Dojindo, Japan), and Annexin V-FITC (A211, Vazyme, China). The oocytes were observed using an Olympus IX81 fluorescence microscope (Olympus IX81-FV1000).

### Targeted lipidomics

Oocytes were dissolved in 500 µl of methanol and sonicated at 67 Hz for 3 min (5 times). The samples were freeze–thawed 3 times with liquid nitrogen during beating. Each sample was then transferred to a 15 ml glass tube, and another 500 µl of methanol was added. Next, 2 ml of chloroform was added, and the mixture was vortexed vigorously for 30–60 s. The sample was spun down for 1 min at 4000 rpm, and the supernatant was transferred to a new 15 ml glass tube. Next, 400 µl of 50 mM citric acid and 800 µl of chloroform were added to the suspension, which was mixed well and spun to achieve phase separation. The upper phase was aspirated, and the bottom phase was separated into 2–3 EP tubes. A SpeedVac was used for concentration. The AB Sciex Triple Quad 6500+ System was used.

### IHC and immunofluorescence

Paraffin-embedded ovarian tissue sections were deparaffinized, immersed in retrieval solution (10 mM sodium citrate), heated in an autoclave, blocked with 3% BSA (G5001, Servicebio), and then incubated overnight with the indicated primary antibodies. The antibodies employed were as follows: anti-NF2 (ab88957, Abcam), anti-active YAP (ab205270, Abcam), anti-transferrin receptor (ab214039, Abcam), anti-GPX4 (A13309, ABclonal), anti-COX2 (12375-1-AP, Proteintech), and anti-HRP secondary antibodies. Staining was performed using Vectastain ABC kits and DAB peroxidase substrate kits (G1211, Servicebio, China).

Ovaries were fixed in 4% paraformaldehyde for 2 h at room temperature, dehydrated with 30% sucrose overnight at room temperature, embedded with O.C.T. (4583, Sakura Finetek, Japan) and frozen-sectioned at a thickness of 10 μm. The ovary sections were dried at 37 °C for 30 min, washed with PBS and then incubated with Nile red (HY-D0718, MCE, USA) at 4 °C overnight. After being washed with PBS, the nuclei were stained using 1 μg/ml DAPI (Sigma). The slides were analyzed using a confocal microscope (Olympus IX81-FV1000).

Isolated oocytes were fixed with 4% paraformaldehyde for 1 hour at room temperature and washed 3 times with 1% BSA in PBS. Then, they were stained with 10 µg/ml Nile red at 4 °C overnight. The nuclei were stained using DAPI (ab228549, Abcam). Oocyte neutral lipids were observed using a confocal microscope (Olympus IX81-FV1000).

### Animal treatments and related measurements

Thirteen-week-old mice were treated with Fer-1 (S7243, Selleck Chemicals, USA) for three weeks. The mice received daily intraperitoneal injections of either Fer-1 (1 mg/kg) or vehicle (corn oil). Then, estrous cycle detection, ovarian follicle counting, and oocyte staining with lipid ROS were performed. Mice at postnatal day 7 were treated with verteporfin (VP) (CL 318952, MCE) at 3-day intervals for 14 days and injected i.p. with either VP (100 mg/kg) or vehicle (corn oil).

### RNA isolation, reverse transcription, and RT–qPCR

Total RNA was extracted with TRIzol reagent (Takara, Japan) according to the manufacturer’s protocol, and an equal amount of RNA from each sample was extracted with a reverse-transcription kit (R233, Vazyme, China). Real-time PCR was performed using SYBR® Green gene expression assays (R711, Vazyme, China). The primers are listed in Supplementary Table 1.

### Western blotting

Tissue and cells were extracted in cold RIPA buffer (BL507A, Biosharp, China), which was supplemented with 1 mM phenylmethylsulfonyl fluoride (P0100, Biosharp) and protease inhibitor cocktail (HY-K0021, MCE). The protein lysates were resolved by SDS–polyacrylamide gel electrophoresis (SDS-PAGE). After protein transfer, the membrane was blocked with 5% skim milk and incubated with antibodies overnight. After three times washes, the membranes were incubated with HRP-conjugated secondary antibody at room temperature, and then washed three times with PBST. The images were captured with a chemiluminescence detection system (Bio-Rad). The antibodies employed were as follows: anti-NF2 (1:1000, ab88957, Abcam), anti-YAP (phospho-S127) (1:1000, ab76252, Abcam), anti-active YAP (1:1000, ab205270, Abcam), anti-YAP (1:1000, sc-101199, Santacruz, USA), anti-transferrin receptor (1:1000, ab214039, Abcam), anti-GPX4 (1:1000, A13309, ABclonal), anti-ACSL4 (1:1000, A20414, ABclonal), anti-β-actin (1:1000, 3700, Cell signaling), anti-P53 (1:1000, M1312-2, HuaBio, China), anti-PARP (1:1000, ET1608-56, HuaBio), anti-BAX (1:1000, ET1603-34, HuaBio), anti-BCL-2 (1:1000, ER1802-97, HuaBio), anti-Caspase3 (1:1000, 66470-2-Ig, Proteintech, USA) and anti-BNC1(1:1000, ARP33283_P050, Aviva, U.K.).

### Luciferase assay

Expression vectors encoding pcDNA3.1-*Bnc1* (mouse), the pGL3-*Nf2* promoter (mouse) and the pGL3-*Nf2*-truncation mutation promoter (mouse) were cotransfected into HEK293T cells using Lipofectamine™ 3000 Transfection Reagent (L3000150, Thermo Fisher). After transfection for 24 h, the cultured cells were collected with passive lysis buffer and measured with the Dual-Luciferase® Reporter Assay System (Promega) according to the manufacturer’s instructions.

### Statistical analysis

Statistical analysis was performed with GraphPad Prism software (GraphPad Software, San Diego, CA). The results are given as the means and standard deviations (SDs). Each experiment included at least three independent samples and was repeated at least three times. Group comparisons were made by two-tailed unpaired Student’s *t* tests. *P* values of <0.05 were considered to indicate statistical significance. The sample sizes (“n”), the statistical tests and the exact *p* values were described in each figure legend.

### Reporting summary

Further information on research design is available in the Nature Research Reporting Summary linked to this article.

## Supplementary information

Supplementary Information

Reporting Summary

## Data Availability

The data supporting the findings from this study are available within the article file and its supplementary information. Sequence data that support the findings of this study has been deposited in the Gene Expression Omnibus database (GEO) repository under accession code, GSE194194, GSE194195, GSE194196. Source data are provided with this paper.

## Source data

Source Data
