# Supplementary material for: BNC1 deficiency-triggered ferroptosis through the NF2-YAP pathway induces primary ovarian insufficiency
Source: Nat Commun. 2022 Oct 5;13:5871. doi: 10.1038/s41467-022-33323-8 (PMC9534854; doi:10.1038/s41467-022-33323-8)

Supplementary Information:

BNC1 deficiency-triggered ferroptosis through the NF2-YAP pathway induces primary ovarian insufficiency

Feixia Wang[1, #], Yifeng Liu[1, #], Feida Ni[1, #], Jiani Jin[1], Yiqing Wu[1], Yun Huang[1], Xiaohang Ye[1], Xilin Shen[2], Yue Ying[1], Jianhua Chen[3], Ruixue Chen[1], Yanye Zhang[1], Xiao Sun[1], Siwen Wang[1,4], Xiao Xu[1], Chuan Chen[1], Jiansheng Guo[5], Dan Zhang[1,6*]

[1]Key Laboratory of Reproductive Genetics (Ministry of Education) and Department of Reproductive Endocrinology, Women's Hospital, Zhejiang University School of Medicine, Zhejiang, 310006, China.

[2]College of Computer Science and Technology, Zhejiang University, Zhejiang, 310027, PR China.

[3]Department of Pathology, Women's Hospital, Zhejiang University School of Medicine, Zhejiang, 310006, People's Republic of China.

[4]Department of Nutrition, Harvard T.H. Chan School of Public Health, Boston, MA, USA, 02215

[5]Center of Cryo-Electron Microscopy, Zhejiang University, Hangzhou, Zhejiang, China.

[6]Clinical Research Center on Birth Defect Prevention and Intervention of Zhejiang Province, Hangzhou, 310006, China.

[#]These authors contributed equally: Feixia Wang, Yifeng Liu, Feida Ni.

[*]email: zhangdan@zju.edu.cn

Supplementary figures

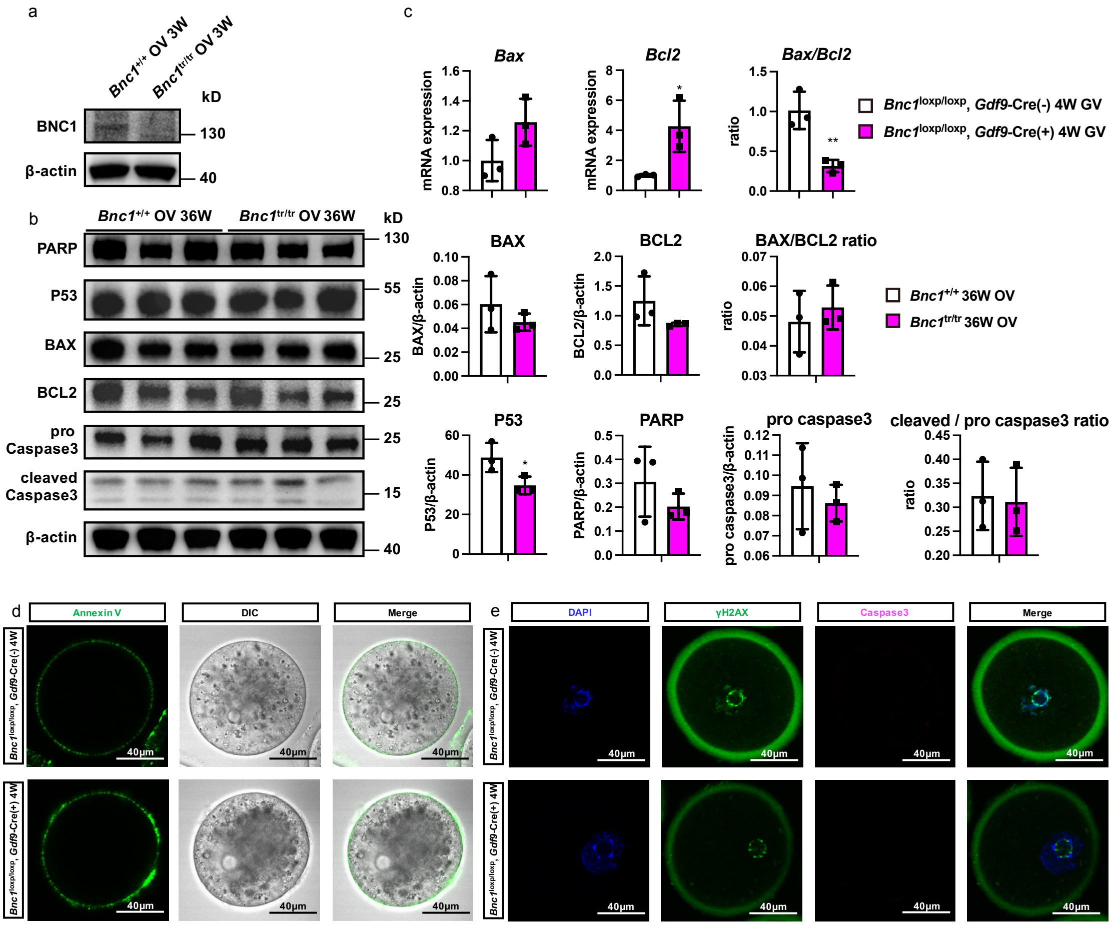

Supplementary Figure 1. Oocyte-specific *Bnc1* knockout induces follicular atresia through nonapoptotic cell death.

a Ovaries from *Bnc1*$^{+/+}$ and *Bnc1*$^{tr/tr}$ mice at 3 weeks old were subjected to WB analysis. The expression levels of BNC1and β-Actin are shown (3 independently experiments). **b** Ovaries from 36-week-old *Bnc1*$^{+/+}$ (n=3) and *Bnc1*$^{tr/tr}$ (n=3) mice were subjected to WB analysis. The expression levels of P53, PARP, Caspase3, cleaved- Caspase3, BAX and BCL2are shown (the error bars indicate the mean values ± SDs, unpaired t test, two-tailed, p value=0.0470 for P53, p value=0.3133 for PARP, p value=0.5635 for Caspase3, p value=0.8399 for cleaved-Caspase3, p value=0.3482 for BAX, p value=0.1716 for BCL2 and p value=0.5513 for BAX/BCL2). **c** GV oocytes from *Bnc1*$^{loxP/loxP}$*, Gdf9*-Cre (-) (n=3) and *Bnc1*$^{loxP/loxP}$*, Gdf9*-Cre (+) (n=3) mice at 4 weeks old were used for RT–PCR. The mRNA expression of *Bax* and *Bcl2* is shown (the error bars indicate the mean values ± SDs, unpaired t test, two-tailed, p value=0.1003 for *Bax*, p value=0.0296 for *Bcl2* and p value=0.0081 for *Bax*/*Bcl2*). **d** GV oocytes obtained from *Bnc1*$^{loxP/loxP}$*, Gdf9*-Cre (-) and *Bnc1*$^{loxP/loxP}$*, Gdf9*-Cre (+) mice at 4 weeks old were used for detection of early apoptosis (3 independent experiments with total oocyte numbers > 30 oocytes). **e** GV oocytes obtained from *Bnc1*$^{loxP/loxP}$*, Gdf9*-Cre (-) and *Bnc1*$^{loxP/loxP}$*, Gdf9*-Cre (+) mice at 4 weeks old were used for detection of Caspase3 (CAS3) and the DNA damage marker γ-H2AX (3 independent experiments with total oocyte numbers > 30 oocytes), *p value < 0.05, **p value < 0.01 and ***p value < 0.001. Source data are provided as a Source Data file.

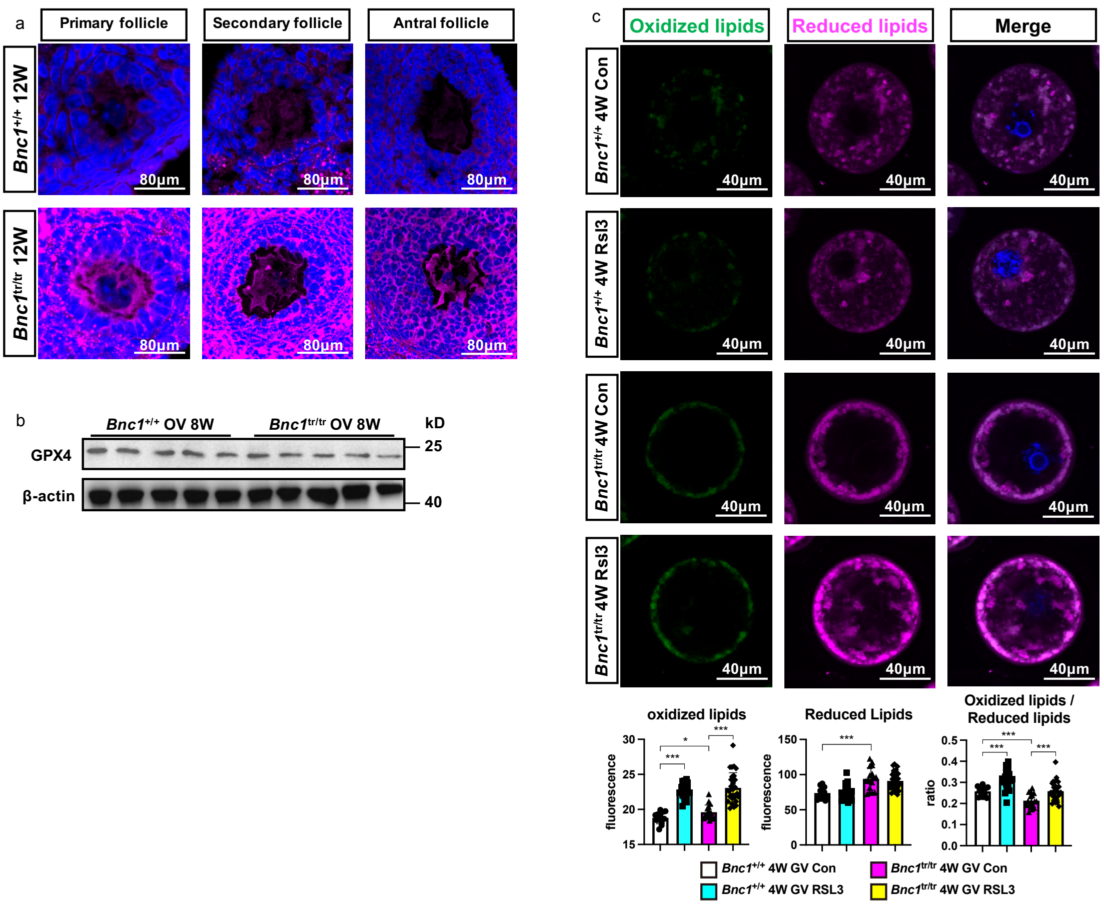

Supplementary Figure 2. Oocytes affected by *Bnc1* mutation are more sensitive to ferroptosis.

a Nile red of of *Bnc1*$^{+/+}$ (n=3) and *Bnc1*$^{tr/tr}$ (n=3) mouse ovaries. **b** WB of GPX4 in mouse ovaries of *Bnc1*$^{+/+}$ (n=5) and *Bnc1*$^{tr/tr}$ (n=5) mouse ovaries. **c** Lipid ROS of GV oocytes after RSL3 treatment (for oxidized lipids p value<0.0001for *Bnc1*$^{+/+}$ RSL3, p value=0.0315 for *Bnc1*$^{tr/tr}$ Con, p value<0.0001 for *Bnc1*$^{tr/tr}$ RSL3, for reduced lipids p value=0.8256 for of *Bnc1*$^{+/+}$ RSL3, p value=0.0004 for *Bnc1*$^{tr/tr}$ Con, p value=0.4840 *Bnc1*$^{tr/tr}$ RSL3, for oxidized/reduced lipids p value=0.0013 for *Bnc1*$^{+/+}$ RSL3, p value=0.0004 for *Bnc1*$^{tr/tr}$ Con and p value=0.0009 for *Bnc1*$^{tr/tr}$ RSL3). The error bars indicate the mean values ± SDs, unpaired t test, two-tailed, 3 independent experiments with total oocyte numbers > 30 oocytes, *p value < 0.05, **p value < 0.01 and ***p value < 0.001. Source data are provided as a Source Data file.

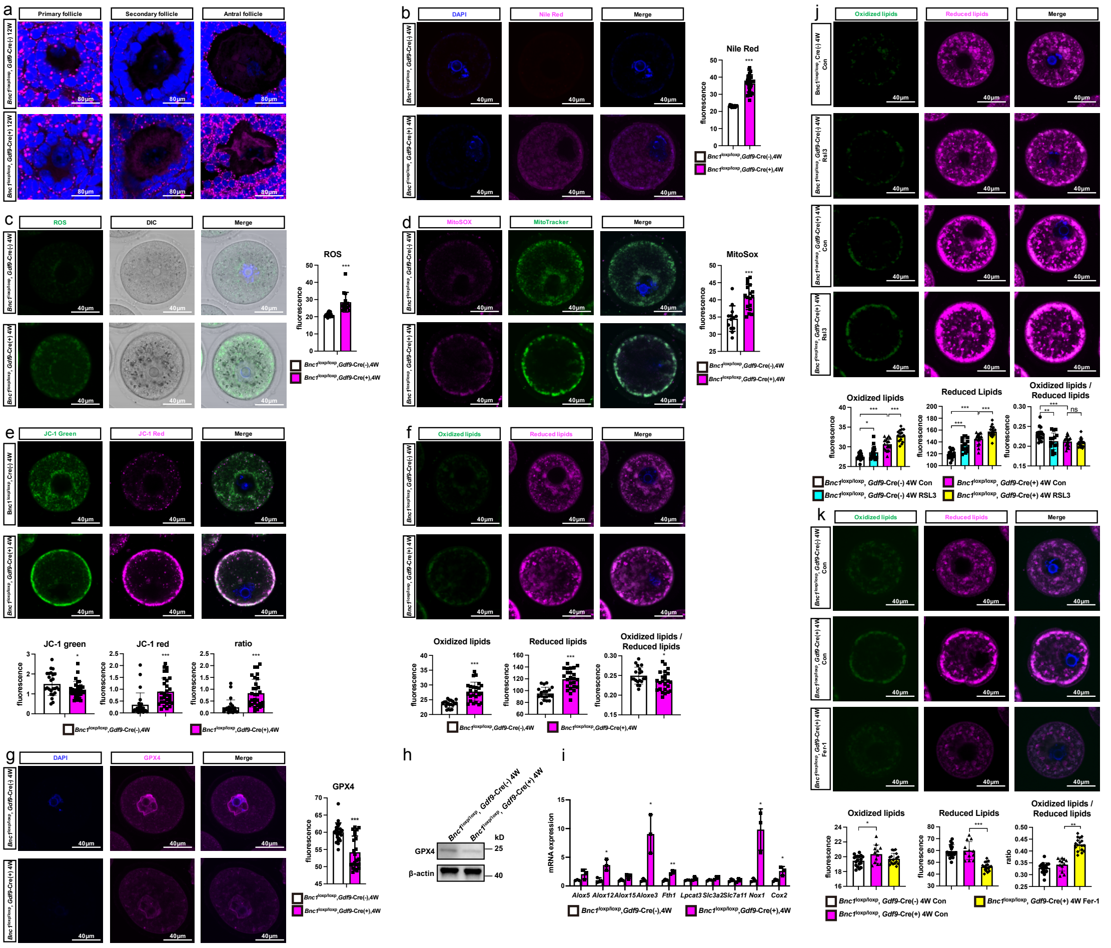

Supplementary Figure 3. Oocyte-specific *Bnc1*-knockout oocytes are more sensitive to ferroptosis.

a Nile red of *Bnc1*$^{loxP/loxP}$, *Gdf9*-Cre (-) and *Bnc1*$^{loxP/loxP}$, *Gdf9*-Cre (+) mouse ovaries (n=3). **b** Nile red of GV oocytes (p value<0.0001). **c** ROS of GV oocytes (p value<0.0001). **d** MitoSOX and MitoTracker in GV oocytes (p value<0.0001). **e** JC-1 of GV oocytes (p value=0.0374 for JC-1 green, p value=0.0004 for JC-1 red and p value<0.0001 for JC-1 red/green). **f** Lipid ROS of GV oocytes (p value<0.0001 for oxidized lipids, p value<0.0001 for reduced lipids and p value=0.0413 for oxidized/reduced lipids). **g** GPX4 in GV oocytes (p value<0.0001). **h** WB of GPX4 in GV oocytes (3 independently experiments). **i** RT–PCR of ferroptosis-associated markers in GV oocytes (n=3) (p value=0.0226 for *Alox5*, p value=0.0132 for *Alox12*, p value=0.0578 for *Aloxe15*, p value= 0.0144 for *Aloxe3*, p value=0.0028 for *Fth1*, p value=0.2300 for *Lpcat3*, p value=0.0691 for *Slc3a2* and p value=0.9711 for *Slc7a11*, p value=0.0267 for *Cox2* and p value=0.0133 for *Nox1*).**j** Lipids ROS in GV oocytes after RSL3 treatment (for oxidized lipids p value=0.0159 for *Bnc1*$^{loxP/loxP}$, *Gdf9*-Cre (-) RSL3, p value<0.0001 for *Bnc1*$^{loxP/loxP}$, *Gdf9*-Cre (+) Con, p value=0.0001 for *Bnc1*$^{loxP/loxP}$, *Gdf9*-Cre (+) RSL3, for reduced lipids p value<0.0001 for *Bnc1*$^{loxP/loxP}$, *Gdf9*-Cre (-) RSL3, p value<0.0001 for *Bnc1*$^{loxP/loxP}$, *Gdf9*-Cre (+) Con, p value=0.0005 for *Bnc1*$^{loxP/loxP}$, *Gdf9*-Cre (+) RSL3, for oxidized/reduced lipids p value=0.0072 for *Bnc1*$^{loxP/loxP}$, *Gdf9*-Cre (-) RSL3, p value=0.0005 for *Bnc1*$^{loxP/loxP}$, *Gdf9*-Cre (+) Con and p value=0.4531 for *Bnc1*$^{loxP/loxP}$, *Gdf9*-Cre (+) RSL3). **k** Lipid ROS in GV oocytes after Fer-1 treatment (for oxidized lipids p value=0.0207 for *Bnc1*$^{loxP/loxP}$, *Gdf9*-Cre (+) Con, p value=0.0989 for *Bnc1*$^{loxP/loxP}$, *Gdf9*-Cre (+) Fer-1, for reduced lipids p value=0.8595 for *Bnc1*$^{loxP/loxP}$, *Gdf9*-Cre (+) Con, p value<0.0001 for *Bnc1*$^{loxP/loxP}$, *Gdf9*-Cre (+) Fer-1, for oxidized/reduced lipids p value=0.1353 for *Bnc1*$^{loxP/loxP}$, *Gdf9*-Cre (+) Con and p value<0.0001 for *Bnc1*$^{loxP/loxP}$, *Gdf9*-Cre (+) Fer-1). The error bars indicate the mean values ± SDs, unpaired t test, two-tailed, 3 independent experiments with total oocyte numbers > 30 oocytes, *p value < 0.05, **p value < 0.01 and ***p value < 0.001. Source data are provided as a Source Data file.

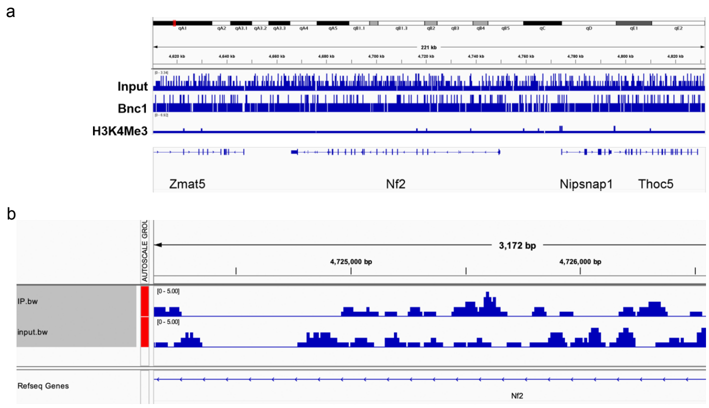

Supplementary Figure 4. ChIP-seq of mouse ovary.

a-b ChIP-seq for BNC1 of mouse ovary and showing the peaks of BNC1 on *Nf2*(n=3).

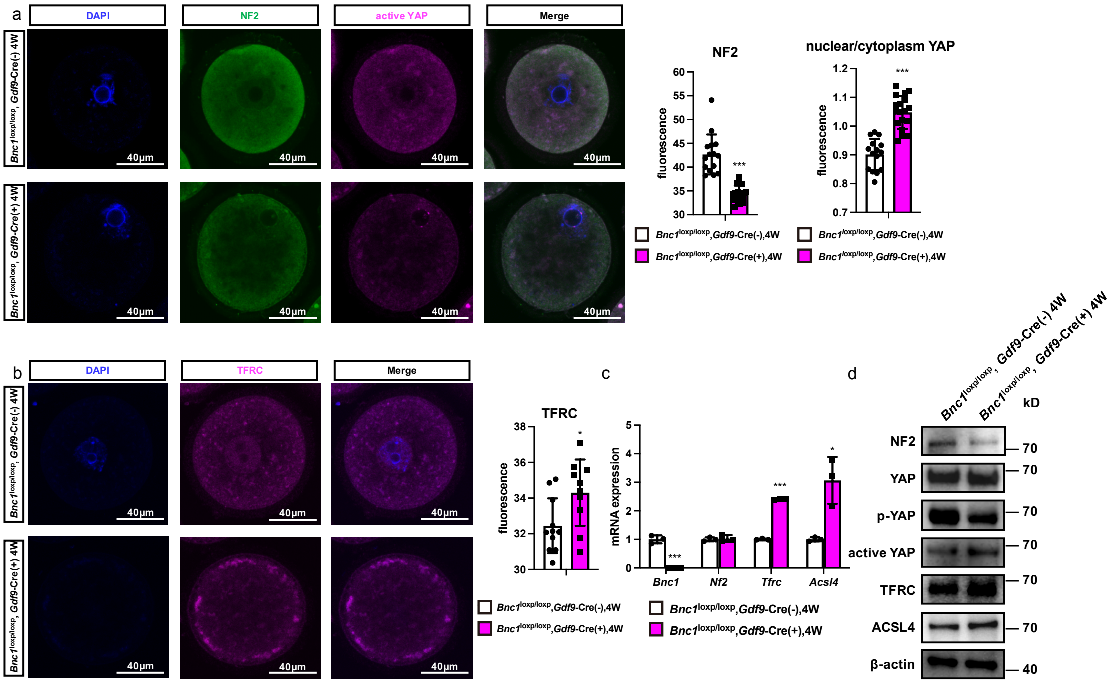

Supplementary Figure 5. Oocyte-specific *Bnc1* knockout sensitizes oocytes to ferroptosis by regulating NF2-YAP signaling.

a NF2 and active YAP of *Bnc1*$^{loxP/loxP}$*, Gdf9*-Cre (-) and *Bnc1*$^{loxP/loxP}$*, Gdf9*-Cre (+) mouse GV oocytes at 4 weeks old (the error bars indicate the mean values ± SDs, unpaired t test, two-tailed, p value<0.0001 for NF2, p value<0.0001 for nuclear/cytoplasmic YAP, 3 independent experiments with total oocyte numbers > 30 oocytes). **b** TFRC staining of *Bnc1*$^{loxP/loxP}$*, Gdf9*-Cre (-) and *Bnc1*$^{loxP/loxP}$*, Gdf9*-Cre (+) mouse GV oocytes at 4 weeks old (the error bars indicate the mean values ± SDs, unpaired t test, two-tailed, p value=0.0213, 3 independent experiments with total oocyte numbers > 30 oocytes). **c** RT–PCR analysis of *Bnc1*, *Nf2*, *Tfrc* and *Acsl4* in *Bnc1*$^{loxP/loxP}$*, Gdf9*-Cre (-) (n=3) and *Bnc1*$^{loxP/loxP}$*, Gdf9*-Cre (+) (n=3) mouse GV oocytes at 4 weeks old (p value=0.0009 for *Bnc1*, p value=0.9150 for *Nf2*, p value<0.0001 for *Tfrc* and p value<0.0001 for *Acsl4,* the error bars indicate the mean values ± SDs, unpaired t test, two-tailed). **d** WB analysis of NF2, p-YAP, active YAP, YAP, ACSL4, TFRC and β-actin in GV oocytes at 4 weeks old (3 independently experiments). *p value < 0.05, **p value < 0.01 and ***p value < 0.001. Source data are provided as a Source Data file.

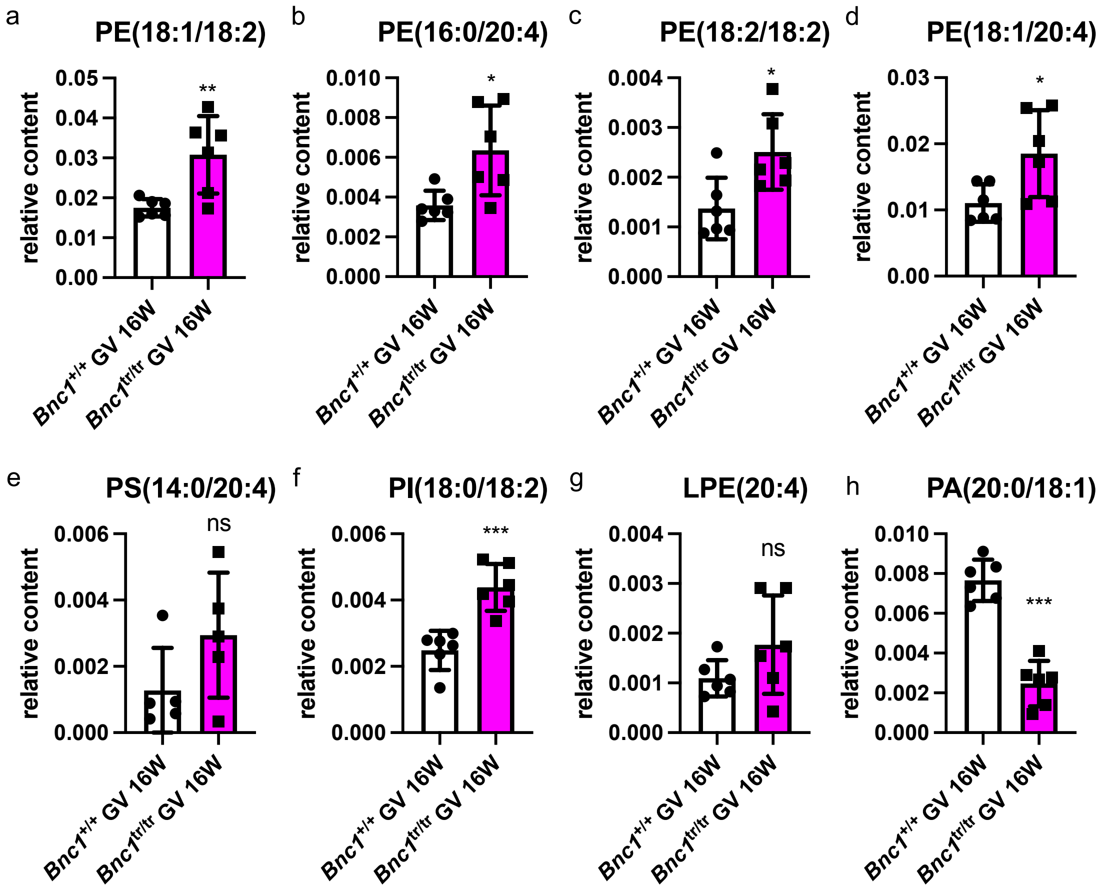

Supplementary Figure 6. Targeted lipidomics of *Bnc1*$^{+/+}$ and *Bnc1*$^{tr/tr}$ GV oocytes.

a Targeted lipidomics showed increased PE (18:1/18:2) (P=0.0084). **b** Targeted lipidomics showed increased PE (16:0/20:4) (P=0.0173). **c** Targeted lipidomics showed increased PE (18:2/18:2) (P=0.0176). **d** Targeted lipidomics showed increased PE (18:1/20:4) (P=0.0283). **e** Targeted lipidomics showed increased PS (14:0/20:4) (P=0.1404). **f** Targeted lipidomics showed increased PI (18:0/18:2) (P=0.0005). **g** Targeted lipidomics showed increased LPE (20:4) levels (P=0.1481). **h** Targeted lipidomics showed decreased PA (20:0/18:1) levels (P<0.0001) in *Bnc1*$^{tr/tr}$ mice (n=6) compared to WT mice (n=6) . the error bars indicate the mean values ± SDs, unpaired t test, two-tailed, *p value < 0.05, **p value < 0.01 and ***p value < 0.001. Source data are provided as a Source Data file.

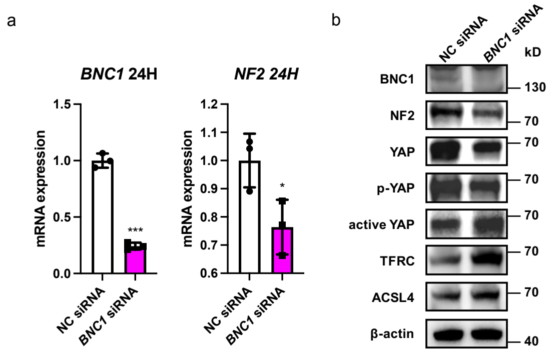

Supplementary Figure 7. ***BNC1*** **regulates NF2-YAP-ferroptosis axis in ES-2 cell line.**

a RT–PCR of *BNC1* and *NF2* after *BNC1* siRNA interference 24 hours in ES-2 cells (the error bars indicate the mean values ± SDs, unpaired t test, two-tailed, p value<0.0001 for *BNC1* and p value=0.0387 for *NF2,* n=3 with 3 independently experiments). **b** WB of BNC1, NF2, p-YAP, active YAP, YAP, TFRC and ACSL4 after *BNC1* siRNA interference 48 hours in ES-2 cells (3 independently experiments). *p value < 0.05, **p value < 0.01 and ***p value < 0.001. Source data are provided as a Source Data file.

Supplementary Tables

Supplement Table 1 RT–PCR Primers and siRNA targeting sequences

		RT–PCR primers and siRNA targeting sequences	
**Gene**	**Species**	**Forward primer (5′–3′)**	**Reverse primer (5′–3′)**
Bax	Mouse	TTGCCCTCTTCTACTTTGCTAG	CCATGATGGTTCTGATCAGCTC
Bcl2	Mouse	GAACTCAAAGAAGGCCACAATC	GATGACTTCTCTCGTCGCTAC
Cox2	Mouse	CATGAGCCGTCCCCTCACTAGG	TGGTCGGTTTGATGCTACTGTTGC
Nox1	Mouse	GTGCCTTTGCCTGGTTCAACAAC	AGCCAGTGAGGAAGAGACGGTAG
Bnc1	Mouse	GTCCTCAGTCACACAGAGTATC	CTCAGTCTCCCTCTCTGAATTG
Nf2	Mouse	GAGGAGAGAATTACTGCTTGGT	CCCTTTTTATTCCGGATTGCAA
Tfrc	Mouse	TCACACTCTCTCAGCTTTAGTG	TGGTTTCTGAAGAGGGTTTCAT
Acsl4	Mouse	CAATAGAGCAGAGTACCCTGAG	TAGAACCACTGGTGTACATGAC
Alox5	Mouse	GGCGAGATCTACCTAGTCAAAA	GATGTGAATTTGGTCATCTCGG
Alox12	Mouse	CAAGGAGGAGGAGTTTGACTTC	GAACTGTGATGAGGTTGCAGAA
Alox15	Mouse	GGAAGAAAGGAGGAGTCTGTAC	GTCTTTTGTCCTCTCGAAATCG
Aloxe3	Mouse	AATCATCTTTAATTGCTCCGCC	GTAACTCTTCATTGTCGTGTCG
Lpcat3	Mouse	CATGAAAGTGTGGCTCTTTGAA	GTTTGAAGATGTAACGGGCTAC
Slc3a2	Mouse	AGGGACTCCTGTTTTTAGCTAC	GTGAAAGATGCTGGACTCATTC
Slc7a11	Mouse	CTATTTTACCACCATCAGTGCG	ATCGGGACTGCTAATGAGAATT
Fth1	Mouse	TAAAGAAACCAGACCGTGATGA	ATTCACACTCTTTTCCAAGTGC
Gapdh	Mouse	CAGGAGGCATTGCTGATGAT	GAAGGCTGGGGCTCATTT
Nf2(ChIP)	Mouse	ACCAGTCTTGCTAGAGTAGG	AGGCCTACTCCACCAATTGA
BNC1	Human	GTCCTCAGTCACACAGAGTATC	CTCAGTCTCCCTCTCTGAATTG
NF2	Human	GAGGAGAGAATTACTGCTTGGT	CCCTTTTTATTCCGGATTGCAA
GAPDH	Human	GGAGCGAGATCCCTCCAAAAT	GGCTGTTGTCATACTTCTCATGG
**siRNA**		**Sense**	**Antisense**
BNC1	Human	GGACACUUCAGGAUUAUAUTT	AUAUAAUCCUGAAGUGUCCTT
Negative control	Human	UUCUUCGAACGUGUCACGUTT	ACGUGACACGUUCGGAGAATT

Uncropped scans of all blots and gels in Supplementary Figures

Supplementary Figure 1a Ovaries from *Bnc1*$^{+/+}$ and *Bnc1*$^{tr/tr}$ mice at 3 weeks old were subjected to WB analysis.

β-Actin

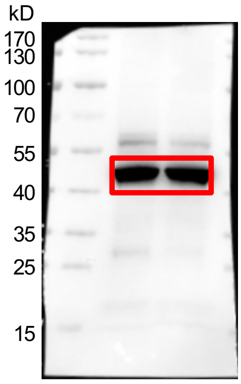

BNC1

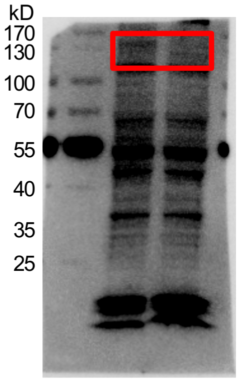

Supplementary Figure 1b Ovaries from 36-week-old *Bnc1*$^{+/+}$ and *Bnc1*$^{tr/tr}$ mice were subjected to WB analysis.

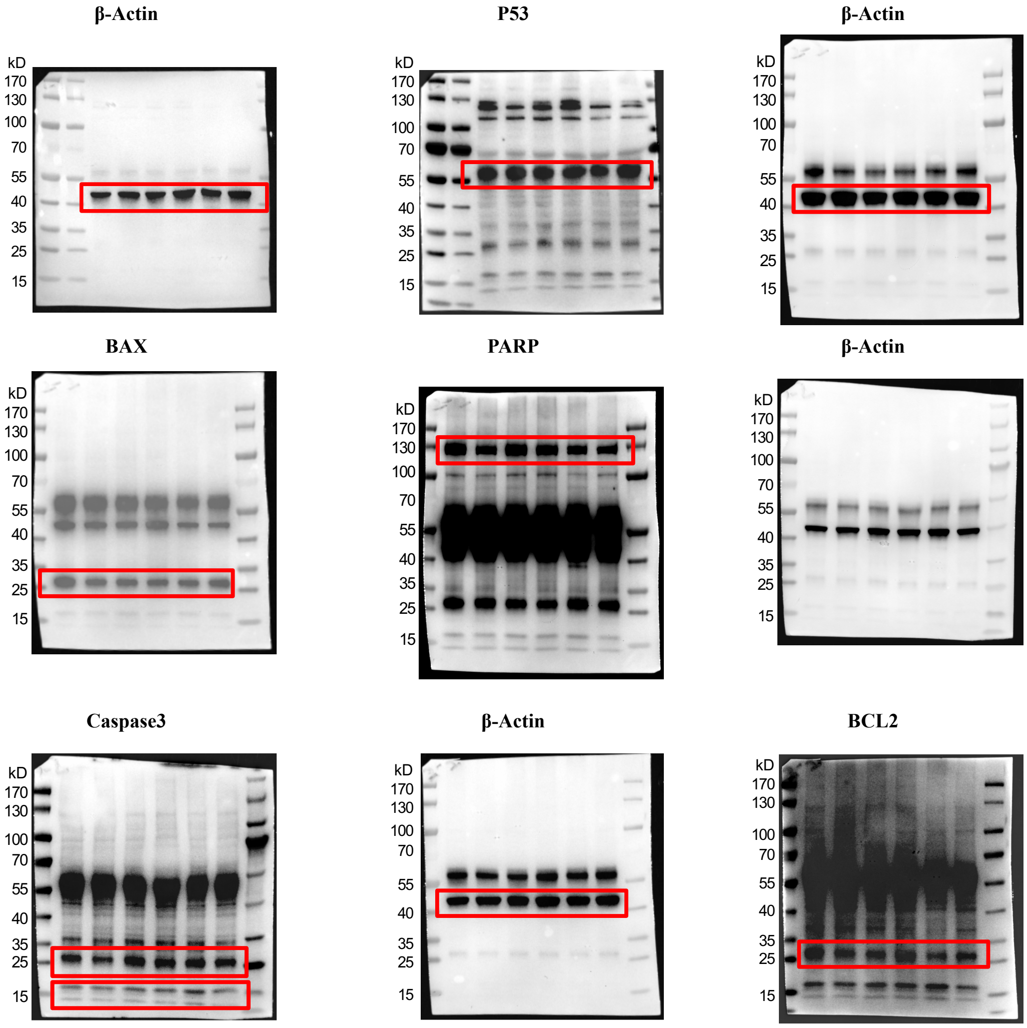

Supplementary Figure 2b WB of GPX4 in mouse ovaries of *Bnc1*$^{+/+}$ and *Bnc1*$^{tr/tr}$ mouse ovaries.

β-Actin

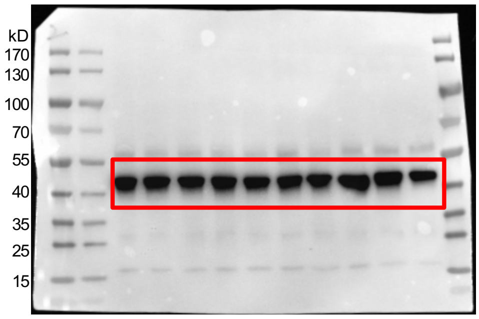

GPX4

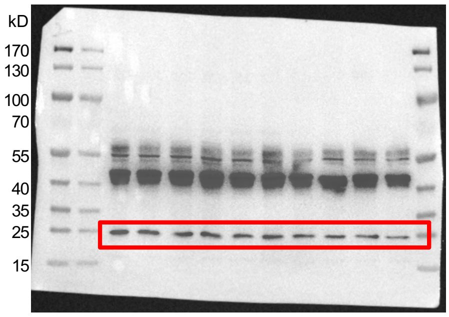

Supplementary Figure 3h WB of GPX4 in GV oocytes.

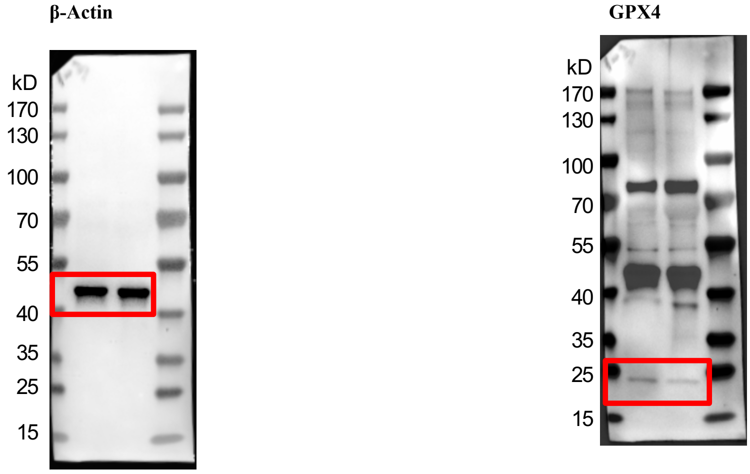

Supplementary Figure 5d WB analysis of NF2, p-YAP, active YAP, YAP, ACSL4, TFRC and β-actin in GV oocytes at 4 weeks old.

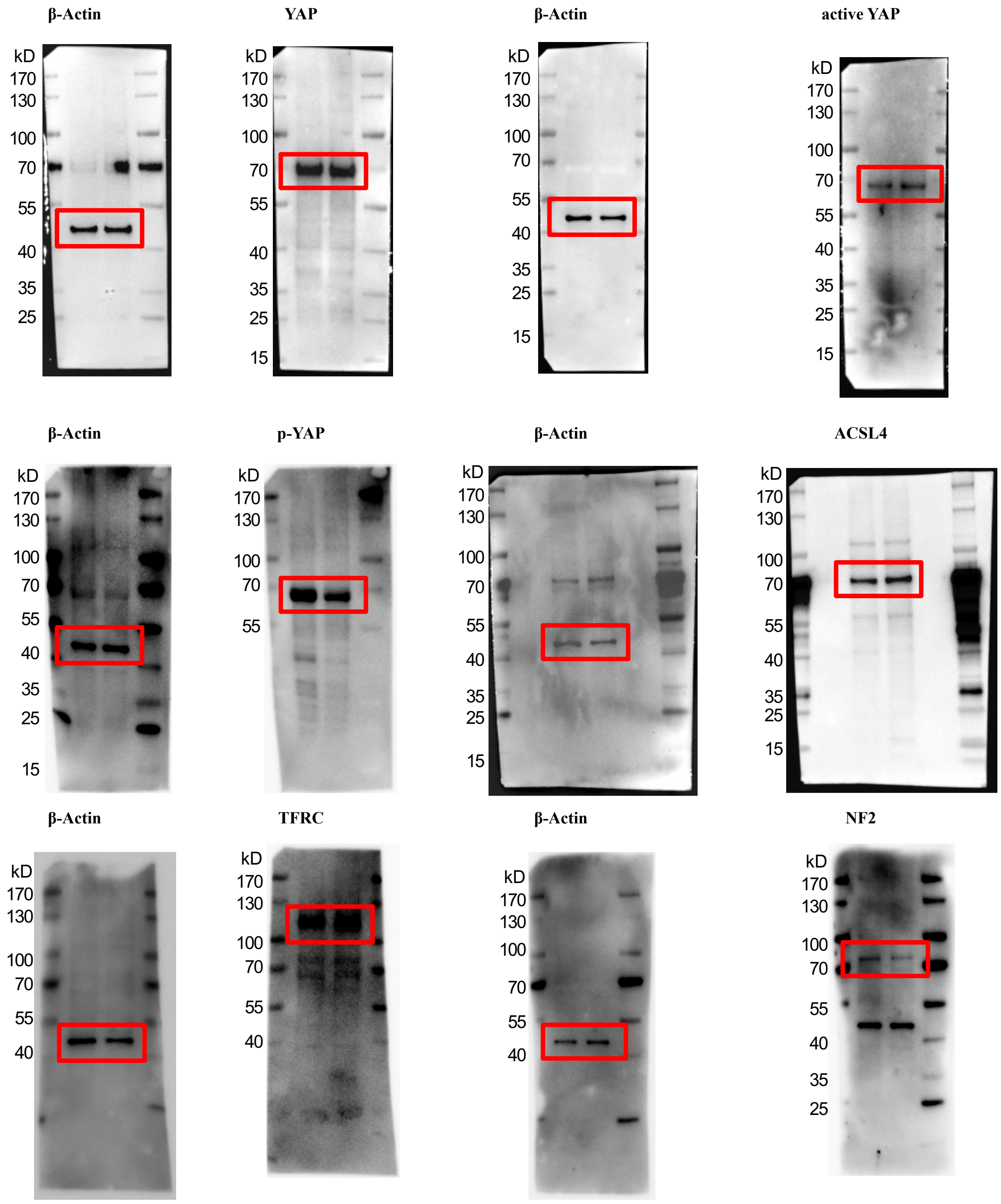

Supplementary Figure 7b WB of BNC1, NF2, p-YAP, active YAP, YAP, TFRC and ACSL4 after *BNC1* siRNA interference 48 hours in ES-2 cells.

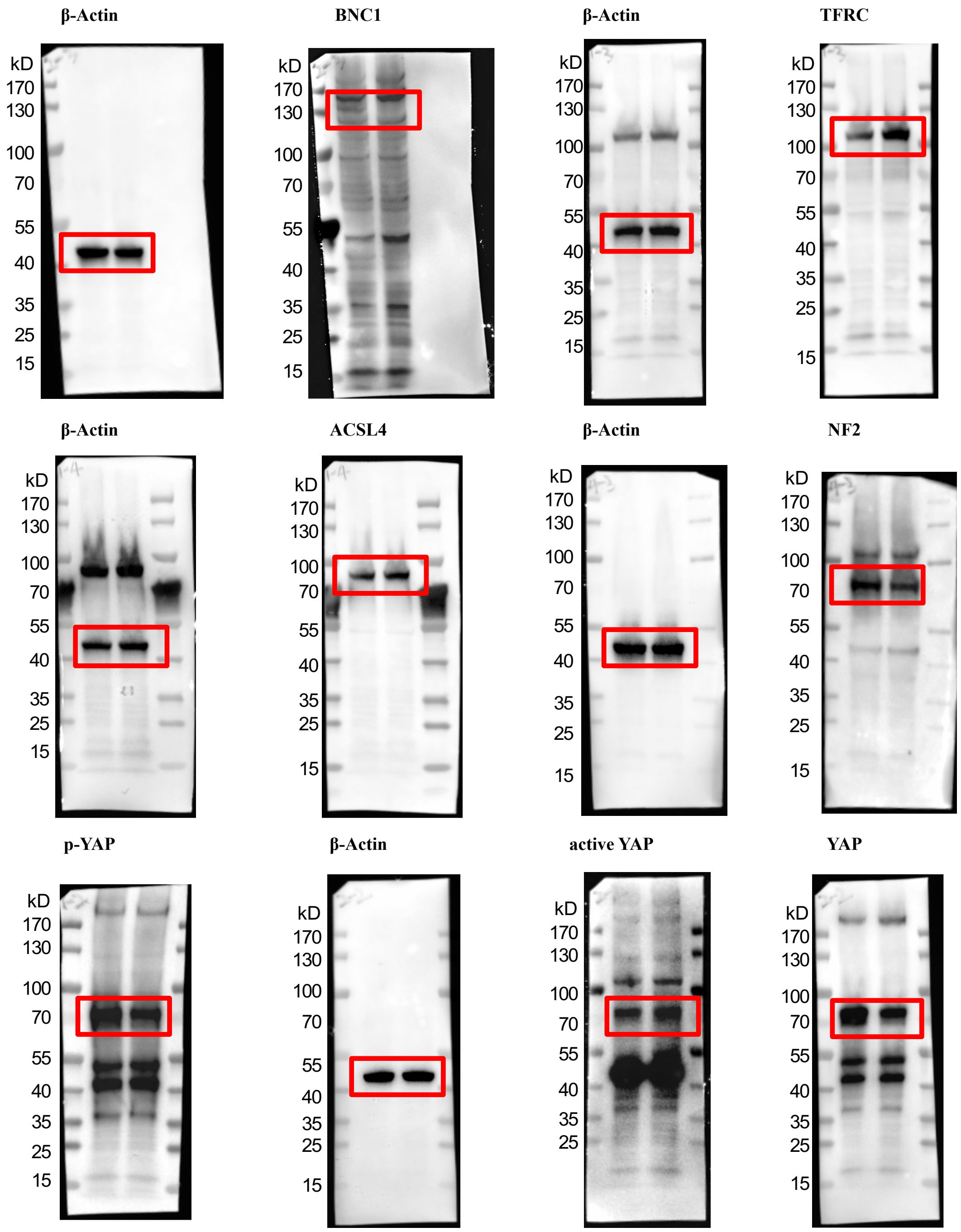

Supplement: Supplementary file 1 — Supplementary Information [file 41467_2022_33323_MOESM1_ESM.pdf]
