# Supplementary material for: BNC1 deficiency-triggered ferroptosis through the NF2-YAP pathway induces primary ovarian insufficiency
Source: Nat Commun. 2022 Oct 5;13:5871. doi: 10.1038/s41467-022-33323-8 (PMC9534854; doi:10.1038/s41467-022-33323-8)
Supplement: Supplementary file 2 — Reporting Summary [file 41467_2022_33323_MOESM2_ESM.pdf]

nature portfolio

Corresponding author(s): Dan Zhang

Last updated by author(s): Aug 31, 2022

Reporting Summary

Nature Portfolio wishes to improve the reproducibility of the work that we publish. This form provides structure for consistency and transparency in reporting. For further information on Nature Portfolio policies, see our Editorial Policies and the Editorial Policy Checklist.

Statistics

For all statistical analyses, confirm that the following items are present in the figure legend, table legend, main text, or Methods section.

n/a	Confirmed	
☐	☒	The exact sample size (*n*) for each experimental group/condition, given as a discrete number and unit of measurement
☐	☒	A statement on whether measurements were taken from distinct samples or whether the same sample was measured repeatedly
☐	☒	The statistical test(s) used AND whether they are one- or two-sided<br>*Only common tests should be described solely by name; describe more complex techniques in the Methods section.*
☒	☐	A description of all covariates tested
☒	☐	A description of any assumptions or corrections, such as tests of normality and adjustment for multiple comparisons
☐	☒	A full description of the statistical parameters including central tendency (e.g. means) or other basic estimates (e.g. regression coefficient) AND variation (e.g. standard deviation) or associated estimates of uncertainty (e.g. confidence intervals)
☐	☒	For null hypothesis testing, the test statistic (e.g. *F*, *t*, *r*) with confidence intervals, effect sizes, degrees of freedom and *P* value noted<br>*Give P values as exact values whenever suitable.*
☒	☐	For Bayesian analysis, information on the choice of priors and Markov chain Monte Carlo settings
☒	☐	For hierarchical and complex designs, identification of the appropriate level for tests and full reporting of outcomes
☒	☐	Estimates of effect sizes (e.g. Cohen's *d*, Pearson's *r*), indicating how they were calculated

Our web collection on statistics for biologists contains articles on many of the points above.

Software and code

Policy information about availability of computer code

Data collection	Western blot images were capatured using Bio-Rad Image Lab 6.1, flurorescent images were acquired using Olympus IX81-FV1000 confocal microscopy and analysed using Image J 1.52K software. qRT-PCR data were collect by Roche and analysed using Microsoft Excel 2016 and Graph Pad Prism 8. IHC images were acquired using Olympus Digital slice Scanner VS200.
Data analysis	Microsoft Excel 2016 and Graph Pad Prism 8 for statistical analysis and graph plotting

For manuscripts utilizing custom algorithms or software that are central to the research but not yet described in published literature, software must be made available to editors and reviewers. We strongly encourage code deposition in a community repository (e.g. GitHub). See the Nature Portfolio guidelines for submitting code & software for further information.

Data

Policy information about availability of data

All manuscripts must include a data availability statement. This statement should provide the following information, where applicable:

- Accession codes, unique identifiers, or web links for publicly available datasets
- A description of any restrictions on data availability
- For clinical datasets or third party data, please ensure that the statement adheres to our policy

ChIP-seq data and RNA-seq data in this study have been deposited in GEO (Gene Expression Omnibus) under accession number GSE194194 (oocyte RNA seq), GSE194195(CHIP-seq), GSE194196(ovary RNA Seq). The datasets generated during and/or analysed during the current study are available in the GEO repository

Field-specific reporting

Please select the one below that is the best fit for your research. If you are not sure, read the appropriate sections before making your selection.

- [x] Life sciences
- [ ] Behavioural & social sciences
- [ ] Ecological, evolutionary & environmental sciences

For a reference copy of the document with all sections, see nature.com/documents/nr-reporting-summary-flat.pdf

Life sciences study design

All studies must disclose on these points even when the disclosure is negative.

Sample size	For the animal experiment, sample size was determined based on previous experience and literature reports [PMID 33953177]. For cell data, we aimed to collected data from three biological replicates when possible.
Data exclusions	No data have been excluded from the analyses
Replication	All in vitro experiments were repeated at least two independent times and all attempts at replication were successful. All in vivo studies were performed two times and the data were reproducible between each study.
Randomization	For in vitro study cells were based on gain or loss of function experiments with appropriate controls. Cells were seeded identically at the onset of the experiments and randomized into the various treatment groups prior to the beginning of treatment protocols. For in vivo studies, mice were allocated into experimental groups randomly.
Blinding	The investigators were blinded to group allocation during data collection and analysis.

Reporting for specific materials, systems and methods

We require information from authors about some types of materials, experimental systems and methods used in many studies. Here, indicate whether each material, system or method listed is relevant to your study. If you are not sure if a list item applies to your research, read the appropriate section before selecting a response.

Materials & experimental systems

n/a	Involved in the study	
	x	Antibodies
	x	Eukaryotic cell lines
x		Palaeontology and archaeology
	x	Animals and other organisms
x		Human research participants
x		Clinical data
x		Dual use research of concern

Methods

n/a	Involved in the study	
	x	ChIP-seq
x		Flow cytometry
x		MRI-based neuroimaging

Antibodies

Antibodies used	anti-PARP(HuaBio; ET1608-56; HN0420); anti-P53(HuaBio; M1312-2; HH0319), anti-BAX(HuaBio; ET1603-34; HN1221), anti-BCL-2 (HuaBio; ER1802-97; HL0507); anti-γH2AX (Sigma–Aldrich, 05-636-I,3135384); anti-Caspase3(proteintech; 66470-2-Ig; 10021291); anti-Caspase3 (Cell Signaling, 9664, 22); anti-BNC1(ARP33283_P050, aviva, QC2807), anti-BNC1(ThermoFisher, PA5-85984,335F0A33), anti-NF2(Abcam; ab88957; GR3313949-6), anti-active YAP (Abcam; ab205270; GR3233758-11), anti-YAP (phospho-S127,Abcam;ab76252), anti-YAP(Santacruz,sc-101199,L0220), anti-TFRC (Abcam; ab214039; GR3235217-13), anti-GPX4 (ABclonal; A13309; 5500003736), anti-ACSL4(ABclonal; A6828; 5500008689), anti-β-Actin(Cell Signaling, 3700, 15), anti-COX2 (Proteintech,12375-1-AP, 00093476)
Validation	anti-PARP(WB; https://www.huabio.com/products/parp-antibody-clone-su03-68-recombinant-monoclonal-et1608-56), anti-P53(WB; https://www.huabio.com/products/p53-antibody-clone-c8-a11-monoclonal-m1312-2), anti-BAX(WB; https://www.huabio.com/products/bax-antibody-clone-sz3-07-recombinant-monoclonal-et1603-34),anti- BCL-2(WB; https://www.huabio.com/products/bcl2-antibody-polyclonal-er1802-97), anti-γH2AX(WB; https://www.sigmaaldrich.cn/CN/zh/product/mm/05636i); anti-Caspase3(IF; https://www.cellsignal.cn/products/primary-antibodies/cleaved-caspase-3-asp175-5a1e-rabbit-mab/9664?site-search-type=Products&N=4294956287&Ntt=9664&fromPage=plp&_requestid=726591),anti- Caspase3(WB; https://www.ptgcn.com/products/CASP3-Antibody-66470-2-Ig.htm); anti-BNC1(WB; https://www.avivasysbio.com/bnc1-antibody-c-terminal-region-arp33283-p050.html); anti-BNC1(ChIP; https://www.thermofisher.cn/cn/zh/antibody/product/BNC1-Antibody-Polyclonal/PA5-85984); anti-NF2(IF,IHC,WB; https://www.abcam.cn/nf2--merlin-antibody-af1g4-ab88957.html); anti-active YAP (IF,IHC,WB; https://www.abcam.cn/active-yap1-antibody-epr19812-ab205270.html); anti-YAP(WB; https://www.abcam.cn/yap1-phospho-s127-antibody-ep1675y-ab76252.html), anti-YAP(WB; https://www.scbt.com/p/yap-antibody-63-7?requestFrom=search),anti- TFRC (IF,IHC,WB; https://www.abcam.cn/transferrin-receptor-antibody-epr20584-ab214039.html); anti-GPX4(IF,IHC,WB; https://

abclonal.com.cn/catalog/A1933); anti-ACSL4(WB; https://abclonal.com.cn/catalog/A6826); anti-β-Actin(WB; https://www.cellsignal.cn/products/primary-antibodies/b-actin-8h10d10-mouse-mab/3700?_=1661599595814&Ntt=3700&tahead=true); anti-COX2(IHC; https://www.ptgcn.com/products/PTGS2-Antibody-12375-1-AP.htm)

Eukaryotic cell lines

Policy information about cell lines

Cell line source(s)	The human ovarian cancer cell line ES-2 (CL-0079, Procell, China); HEK293T(ATCC, CRL-1573, USA)
Authentication	STR identification
Mycoplasma contamination	All cell lines tested negative for mycoplasma contamination
Commonly misidentified lines (See ICLAC register)	No commonly misidentified cell lines were used in the study.

Animals and other organisms

Policy information about studies involving animals; ARRIVE guidelines recommended for reporting animal research

Laboratory animals	Mus musculus; C57BL/6 J; female; postnatal day1 (PD1) and week 3, 4, 12, 16, 36.The mice were housed under controlled environmental conditions with free access to water and food. The mice were reared in standard conditions with controlled temperature (21-25℃), humidity (40-70%) and 12/12-hour dark/light cycle. All comparisons were made between littermates.
Wild animals	The study did not involve wild animals.
Field-collected samples	The study did not involve samples collected from the fields.
Ethics oversight	The experimental procedure for mice were in accordance with the Institutional Guide of the Animal Care and Use Committee (ACUC) and were approved by the ACUC of the Zhejiang University School of Medicine.

Note that full information on the approval of the study protocol must also be provided in the manuscript.

ChIP-seq

Data deposition

- [x] Confirm that both raw and final processed data have been deposited in a public database such as GEO.
- [x] Confirm that you have deposited or provided access to graph files (e.g. BED files) for the called peaks.

Data access links *May remain private before publication.*	https://www.ncbi.nlm.nih.gov/geo/query/acc.cgi?acc=GSE194195Enter
Files in database submission	H3K4me3.peaks.txt; BNC1.peaks.txt; H3K4me3.bw; input.bw; BNC1.bw
Genome browser session (e.g. UCSC)	not applicable

Methodology

Replicates	1
Sequencing depth	40-50M/sample
Antibodies	H3K4me3(Abcam, ab8580); BNC1(Huaan)
Peak calling parameters	peaks were called using MACS(version 2.1.0).A q-value threshold of 0.05 was used for all data sets. After peak calling, the distribution of chromosome distribution, peak width, fold enrichment, significant level and peak summit number per peak were all displayed.
Data quality	filtered the low quality reads whose N content >=15% and mean quality score <=30. Meanwhile it removed the adapter at the 3' end of read and filtered the reads whose length <50 after trimed.Filtered reads were mapped to the genome by Bowtie2.
Software	Raw reads were first pre-processed by skewer(Version 0.2.2)
